# Hex-3(Z)-enyl butyrate: a key volatile compound conferring resistance against Southeast Asian Thrips (*Thrips parvispinus*) in *Capsicum* spp

**DOI:** 10.1093/hr/uhaf346

**Published:** 2026-01-13

**Authors:** Pavani Narigapalli, Shivashankara Kodthala Seetharamaiah, Prasannakumar Nagalapura Ramakrishnappa, Lakshmana Reddy Dhoranapalli ChinnappaReddy, Kamala Jayanthi Pagadala Damodharam, Vasanthi Raguru Pandu, Reddi Sekhar Managala, Naresh Ponnam, Madhavi Reddy Kambham

**Affiliations:** Division of Vegetable Crops, ICAR—Indian Institute of Horticultural Research, Bengaluru 560089, India; Department of Genetics and Plant Breeding, S.V. Agricultural College, Acharya N.G. Ranga Agricultural University, Tirupati 517502, India; Division of Basic Sciences, ICAR—Indian Institute of Horticultural Research, Bengaluru 560089, India; Division of Crop Protection, ICAR—Indian Institute of Horticultural Research, Bengaluru 560089, India; Division of Basic Sciences, ICAR—Indian Institute of Horticultural Research, Bengaluru 560089, India; Division of Crop Protection, ICAR—Indian Institute of Horticultural Research, Bengaluru 560089, India; Department of Genetics and Plant Breeding, S.V. Agricultural College, Acharya N.G. Ranga Agricultural University, Tirupati 517502, India; Department of Genetics and Plant Breeding, S.V. Agricultural College, Acharya N.G. Ranga Agricultural University, Tirupati 517502, India; Division of Vegetable Crops, ICAR—Indian Institute of Horticultural Research, Bengaluru 560089, India; Division of Vegetable Crops, ICAR—Indian Institute of Horticultural Research, Bengaluru 560089, India

## Abstract

Chilli incurs substantial yield losses due to *Thrips parvispinus* (Karny) infestation, necessitating sustainable resistance breeding strategies. Understanding biochemical basis of resistance will help in exploring the candidate metabolites for indirect selection. LC–MS and GC–MS profiling of two resistant (IIHR-B-HP-79, IIHR 4550) and two susceptible (IIHR 3455, IIHR 4604) chilli accessions were performed. LC–MS profiling revealed Inositol with higher levels in susceptible accession IIHR 3455 (8.74 and 0.33 μg/g; VIP score: 2 and 2.5 under control and infested conditions respectively), indicating its role as a stress-induced metabolite rather than a marker for resistance. Secondary metabolites contribution to resistance was genotype-specific and may possibly be driven by complex interactions among these metabolites. Untargeted leaf volatile profiling revealed Hex-3(Z)-enyl butyrate as a significant volatile compound in the resistant accessions IIHR 4550 and IIHR-B-HP-79. Its high accumulation across two different species suggests that its production is not strictly species-specific. Validation of Hex-3(Z)-enyl butyrate through bioassays and olfactometer studies demonstrated reduced scraping damage percentage at 8 and 16 µL^-L^ concentrations in leaf dip bioassays. Four-arm olfactometer studies indicated that Hex-3(Z)-enyl butyrate significantly affected *T. parvispinus* time spent and entries at 16 µL^-L^. Identified metabolites defences can serve as markers for breeding and also can be explored in pest management strategies.

## Introduction

Chilli (*Capsicum* spp.) is an economically significant crop, widely cultivated for its culinary and various industrial applications. Over the last 20 years, global dry chilli production has exponentially grown and doubled from 2.46 to about 4.15 mT. In India, dry chilli cultivated on 852 000 ha, producing 2.78 mMT with an average yield of 3.37 MT/ha (FAOSTAT, 2023; www.fao.org/ FAOSTAT). However, the yield and quality of chilli are severely threatened by biotic stresses, among which *Thrips parvispinus* (Karny) (Thysanoptera; Thripidae) has emerged as a major pest of concern in India [1]. According to recent estimates, *T. parvispinus* infestations have resulted in crop losses ranging from 30% to 50% in severely affected regions, further emphasizing the need for sustainable and integrated pest management strategies [2–4]. This pest is difficult to manage due to its rapid reproductive cycle, high mobility, and ability to develop resistance to chemical insecticides. *Thrips parvispinus* scrapes on plant attacking leaves, flowers and fruits causing severe economic losses [5]. It causes severe foliage damage leading to complete leaves bronzing, poor growth, flower drop, and deformed fruits [6, 7]. Conventional control strategies relying heavily on chemical pesticides have proven increasingly ineffective and unsustainable, leading to concerns over environmental contamination, pesticide residues in food, and the development of pesticide resistance in insects. As a result, deploying resistant/tolerant varieties has become a key focus in integrated pest management (IPM) strategies. Identifying and developing chilli cultivars with inherent resistance to *T. parvispinus* offers a long-term solution to minimize crop damage and reduce the reliance on chemical interventions.

Plant resistance to insect herbivores is often mediated by a complex array of biochemical compounds including sugars, flavonoids, phenols, secondary metabolites, leaf volatiles, glandular trichomes, and epicuticular waxes [8–10]. These compounds play key roles in both direct and indirect defence mechanisms, acting as deterrents or toxins to herbivores and as attractants to natural enemies of pests [11]. Secondary metabolites, such as phenolic acids and flavonoids, have been shown to contribute significantly to plant defence by inhibiting insect growth and development, deterring feeding, and enhancing oxidative stress responses [12]. Similarly, epicuticular waxes have been implicated in creating physical barriers that deter pest colonization and feeding [13]. Epicuticular wax composition and structure significantly influences pest interactions by affecting adhesion and feeding behaviour [14] in crops. Metabolomics, combined with bioassays provides a rapid and precise method to identify specific biochemical markers associated with resistance for indirect selection. For instance, LC–MS-based metabolomics approaches have identified specific compounds, such as dimer acyclic diterpene glycosides in chilli, which play a role in thrips resistance [15]. GC–MS-based analyses have further revealed that epicuticular wax components, such as alkanes and aldehydes, contribute to resistance by influencing pest behaviour [16]. Further identified metabolites conferring resistance can be explored in development of integrated pest management strategies.

Against southeast Asian thrips, 481 chilli germplasm accessions including species *Capsicum chinense, Capsicum frutescens, Capsicum annuum*, and *Capsicum baccatum* were screened and we identified two promising accessions (IIHR-B-HP-79, IIHR 4550) exhibiting significant resistance to *T. parvispinus* infestation through a multi-stage screening involving field, polyhouse and laboratory evaluations. *Thrips parvispinus* life cycle studies on the susceptible and resistant accessions indicated antibiosis type of resistance in IIHR-B-HP-79 accession as observed by malformed larval and neonates and detrimental effects on lifecycle when nymphs were force fed on IIHR-B-HP-79 leaves. This clearly indicated that the presence of specific metabolic/biochemical compounds in resistant accessions to confer resistance against thrips [1]. Further understanding the biochemical basis of resistance particularly the role of metabolic factors will help in exploring the key associated metabolites as markers for indirect selection in screening for resistance and also in development of integrated pest management strategies. In this direction, through LC–MS, we profiled sugars, flavonoids and phenols, while GC–MS was employed to analyze epicuticular waxes and leaf volatiles in resistant and susceptible accessions under both control and infested conditions to explore key metabolites conferring resistance. The study aimed to identify metabolic signatures that distinguish resistant accessions, offering insights into the biochemical basis of resistance for breeding programs and also in developing IPM strategies.

## Materials and methods

### Plant material and experiment conditions

Two resistant [IIHR-B-HP-79 (*C. frutescens*), IIHR 4550 (*C. chinense*)] and two susceptible chilli accessions [IIHR 3455(*C. annum*), IIHR 4604 (*C. annum*)], previously identified [1] were used in the study. Three replicates per treatment were grown in a completely randomized block design. Each genotype was initially sown in a 50-cell seedling trays and then transplanted into pots measuring 6.5 cm x 7 cm, 45 days after sowing (DAS). After transplanting, both healthy and infested plants were kept in cages inside a polyhouse to prevent other infestations. *T. parvispinus* were inoculated on to plants at 10 days after transplanting (DAT). After the infestation, the leaves were collected from the top 3rd node where the infestation was very high for sample analysis. 

### *Thrips parvispinus* culture

*Thrips parvispinus* culture was maintained on haricot beans (*Phaselous vulgaris*) [1] grown under insect and pesticide free conditions. Female adults were collected from field and were released on beans and kept in plastic boxes at 25 ± 3°C, 75% to 80% RH. Nymphs were continuously transferred on to fresh beans after their emergence. The beans were regularly replaced at every 2 to 3 days to maintain the active culture.

### Trichome analysis and cuticle thickness

Trichomes and cuticle thickness in resistant and susceptible chilli accessions were analysed through SEM imaging to understand trichome type and density. Observations were recorded on the abaxial surface of the leaf, as *T. parvispinus* nymphs and adults predominantly inhabit the lower leaf surface.

### LC–MS profiling of phenols, flavonoids, and sugars

Phenolic compounds and flavonoids were extracted from resistant (IIHR-B-HP-79 and IIHR 4550) and susceptible (IIHR 3455 and IIHR 4604) accessions under control and infested conditions. Leaf samples (2 g) were homogenized in 80% methanol, centrifuged and adjusted to 20 ml. The ethyl acetate extract was hydrolyzed with 2 N NaOH, acidified to pH 2.0, re-extracted with ethyl acetate and evaporated. The residue was dissolved in 1 ml MS-grade methanol, filtered and analyzed using LC–MS/MS with a BEH-C18 column and a mobile phase of 0.1% formic acid in water (solvent A) and 0.2% formic acid in methanol (solvent B) [17, 18]. Sugars were extracted by treating 2 g of sample with warm 80% ethanol, collecting supernatants and concentrating the extract. The ethanol extract was dried and the residue was reconstituted in a 1:1 mixture of solvent A (80:20 acetonitrile: water) and solvent B (30:70 acetonitrile: water with 0.1% ammonium hydroxide) and analysed [2] using a Waters UPLC H-Class system with TQD MS/MS [19].

### GC–MS/MS profiling of epicuticular wax and leaf volatiles

Cuticular wax compounds were extracted by dipping 2 g of leaves in chloroform for 15 seconds. The chloroform extract was evaporated, residue was dissolved in hexane and filtered. An aliquot of 1 μl was analysed through Shimadzu Nexis GC-2030 with a VF-WAX-MS column and TQ 8040 NX detector. Compounds were identified using FFNSC and NIST-2020 spectral libraries.

Volatile compounds were extracted via solid-phase micro extraction (SPME) using a 2 cm fused silica fiber coated with PDMS/DVB/CAR (50/30 μm) and conditioned at 280°C for 1 h. Four grams of leaves were rolled with glass rod to release volatiles and placed in a sealed 100 ml conical flask. The SPME fiber was inserted and exposed to the headspace for 2 h. Subsequently, the fiber was injected into a Shimadzu Nexis GC-2030 coupled with TQ 8040 NX for analysis. Volatiles were identified by comparing masses with FFNSC and NIST-2020 libraries and individual compounds were expressed as relative percent area.

### Leaf dip bioassay and four arm olfactometer bioassay

A leaf disc bioassay was conducted using leaves treated with volatiles and distilled water as solvent. The test concentration range (0.5–16 μl^-l^) was developed based on semi-quantitative GC–MS profiling of epicuticular wax/volatile extracts from resistant accessions. To evaluate dose-dependent responses, we initiated serial dilutions starting from 0.5 μl^-l^, creating a gradient of five concentrations (0.5, 2, 4, 8, and 16 μl^-l^). This approach allowed us to simulate relative biological exposure and assess behavioural effects across a range of physiologically relevant concentrations. This range was used consistently across both leaf dip bioassays and four-arm olfactometer assays to compare behavioural and contact responses of *T. parvispinus*. Treated leaves were dried and placed on 1.5% agar medium and 10 nymphs of *T. parvispinus* were released onto each disc. Each concentration was replicated thrice and scraping damage was quantified using ImageJ software [20] after 72 h. Four-arm olfactometer test was employed to validate the bioassay results and assess *T. parvispinus* behavioural responses to plant volatiles under controlled conditions (27 ± 1°C, diffused lighting, black walls). Behavioural assays were conducted following the procedure described by Kamala Jayanthi *et al.* [21]. A single adult female *T. parvispinus* was released into the central chamber of a four-arm olfactometer and allowed to acclimate for 2 min before observations were recorded for 10 min. The airflow was maintained at 350 ml/min, with one arm containing 10 μl of the plant volatile of interest applied to filter paper, while the remaining three arms received 10 μl of distilled water as solvent blanks. Distilled water was used as a neutral solvent to minimize any potential influence on thrips behaviour. The apparatus was rotated every 2 min to eliminate positional bias, and 20 replicates were performed. Time spent and the number of entries into each arm were recorded using Olfa software (OLFA, Udine, Italy). The inclusion of water-only controls ensured that the behavioural responses observed were attributable to the plant volatiles and not due to solvent effects.

### Data analysis

Statistical analysis was conducted using Metaboanalyst 6.0 software [22] and IBM SPSS 29.0.1.0 [23] software. All the data in LC–MS, GC–MS profiling was normalized, log transformed and pareto scaled in the Metaboanalyst 6.0 software. The original mean values, p value significance, FDR ratio are presented in Tables S1–S3. The original and normalized mean values with the standard errors are provided in Tables S4–S6. The Variable Importance in Projection (VIP) score was calculated using Partial Least Squares Regression (PLSR) to identify key metabolites associated with *T. parvispinus* resistance in chilli. VIP scores were computed using a formula that incorporates the weights and scores of variables across all components of the PLSR model, with VIP > 1 indicating significant contributions to the model. This threshold highlights metabolites that are biologically and statistically relevant, as they contribute substantially to explaining the observed variation in resistance or susceptibility. For further validation studies, only those metabolites with VIP > 1 that also showed consistent and elevated expression in resistant accessions were selected, ensuring a focus on compounds most likely to be associated with resistance traits. Variables with VIP scores below 1 were considered less important and excluded from further analysis to reduce dimensionality and focus on impactful traits. This approach ensures the identification of critical metabolites for resistance breeding while eliminating noise and less relevant contributors. VIP scores are calculated from Partial Least Squares Discriminant Analysis in Metaboanalyst 6.0 software. PLS-DA score plot and K-Means clustering was calculated from Metaboanalyst 6.0 software. The IBM SPSS 29.0.1.0 was used for ANOVA and post-hoc tests. Leaf dip bioassay scraping damage % (cm^2^) was quantified using Image J software [24].

## Results

### Trichomes and cuticular thickness

**Figure 1:**
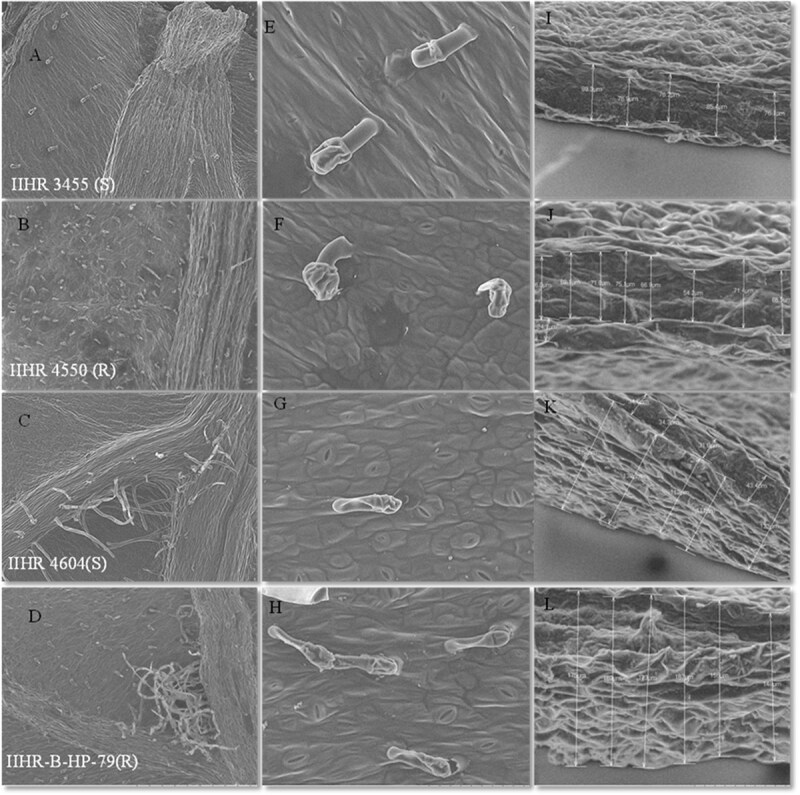
Scanning electron micrographs of the abaxial surface. (a) Panels A, B, C, D have magnification of 100×, with a bar representing 1 mm. (b) Panels E, F, G, H have a magnification of 800×, with a bar representing 100 μm. (c) Panels I, J, K, L are representing cuticle thickness of the leaf which has magnification of 1200× with a bar representing 200 μm. Images from left to right are indicating the trichome count, trichome count, and epicuticular thickness of the same accession. In the above image (S) represents susceptible accession and (R) represents resistant accession.

The trichomes observed across all the accessions were non-glandular trichomes on the abaxial leaf surface. Despite variation in trichome density ranging from 12.80 ± 2.76 in IIHR 3455, 26.20 ± 4.33 in IIHR 4604 and 37.60 ± 4.93 in IIHR-B-HP-79 to a maximum of 60.00 ± 4.59 in IIHR 4550; none of the accessions exhibited glandular trichomes (Table 1, Fig. 1). The resistant accession IIHR-B-HP-79 exhibited the thickest cuticle layer (148.0–189.3 μm), followed by IIHR 4604 (113.0–183.0 μm), IIHR 3455 (76.0–89.3 μm) and IIHR 4550 (54.0–88.5 μm) (Table 1, Fig. 1). Interestingly, both resistant and susceptible accessions showed high cuticle thickness, indicating that cuticular thickness alone may not fully explain thrips resistance, instead, qualitative differences in wax composition appear more critical.

**Table 1:** Trichome count and trichome type in resistant and susceptible accessions

**Accession**	**Trichome count Mean ± SEM**	**Trichome type**	**Cuticular thickness (μm)**
IIHR 3455(S) (Abaxial)	12.80 ± 2.76^b^	Non glandular	76.0–89.3
IIHR 4550 (R) (Abaxial)	60.00 ± 4.59^a^	Non glandular	54.0–88.5
IIHR 4604(S) (Abaxial)	26.20 ± 4.33^b^	Non glandular	113.0–183.0
IIHR-B-HP-79(R) (Abaxial)	37.60 ± 4.93^b^	Non glandular	148.0–189.3

Values followed by the same letter(s) within a column are not significantly different according to Duncan's Multiple Range Test (DMRT) at *P* ≤ 0.05.

### LC–MS profiling of sugars, flavonoids and phenols

The study evaluated the targeted LC–MS profiling of sugars, flavonoids and phenolic acids in susceptible (IIHR 3455 and IIHR 4604; corresponding to K and B in the VIP score table) and resistant (IIHR 4550 and IIHR-B-HP-79; corresponding to A and I in the VIP score table) chilli accessions under control and infested conditions (Figs S1–S3).The primary focus was on compounds with VIP scores >1, indicating their significance in *T. parvispinus* resistance, supported by *P* values <0.05 and trends from the mean values of control and infested plants. Targeted LC–MS profiling of sugars, flavonoids and phenolic acids did not yield any metabolites that consistently correlated with resistance to *T. parvispinus*. Although several compounds showed significant differences in concentration under control or infested conditions, none satisfied the combined criteria of VIP score >1 and consistent elevation in both resistant accessions (IIHR 4550 and IIHR-B-HP-79).The metabolite analysis revealed significant differences in sugar profiling between control and infested conditions in resistant (IIHR-B-HP-79 [I] and IIHR 4550 [A]) and susceptible (IIHR 4604 [B] and IIHR 3455 [K]) accessions against *T. parvispinus*. Across accessions, all quantified sugars showed significant variation under control conditions, with concentration ranging from 0.30–0.67 μg/g for arabinose, 144.47–3596.10 μg/g for fructose, 0.06–0.55 μg/g for fucose, 135.15–447.76 μg/g for glucose, 0.10–8.74 μg/g (VIP score 2.0) for inositol, 4.84–63.91 μg/g (VIP score 1.0–1.5) for maltose, 13.64–74.95 μg/g for mannose, 0.03–0.47 μg/g for rhamnose, 36.07–75.43 μg/g for ribose, 1.16–14.45 μg/g (VIP score 1.0–1.5) for sorbitol, 0.14–0.34 μg/g (VIP score 1.0–1.5) for sucrose and 0 μg/g for xylose. Although several sugars were statistically different among accessions, the concentration ranges of susceptible accessions consistently overlapped with those of resistant accessions, indicating no distinct constitutive difference.

Following infestation, the concentration ranges remained broadly comparable between resistance groups. The observed ranges were 0.24 to 0.35 μg/g (VIP score 1.0–1.5) for arabinose, 162.91 to 711.52 μg/g for fructose, 0.11 to 0.80 μg/g for fucose, 117.01 to 414.63 μg/g for glucose, 0.00 to 0.33 μg/g (VIP score 2.5) for inositol, 1.04 to 24.55 μg/g for maltose, 2.39 to 15.55 μg/g (VIP score 1.0–1.5) for mannose, 0.12 to 0.66 μg/g for rhamnose, 28.01 to 197.70 μg/g for ribose, 2.73 to 7.69 μg/g for sorbitol, 0.29 to 2.75 μg/g for sucrose, and 0 μg/g for xylose. Notably, inositol showed a reduction only within one susceptible genotype, whereas its levels in the other susceptible genotype were identical to those of the resistant accession IIHR-B-HP 79 (0.00 μg/g). For all other sugars, the ranges in susceptible accessions were at par (Heat map in supplementary figures shows the relative abundance of compounds) with resistant accessions, demonstrating no consistent metabolic differentiation following infestation (Table S1, Fig. S1).

Flavonoid concentrations including apigenin, catechin, epicatechin, epigallocatechin, eriodictyol, fisetin, galangin, hesperetin, kaempferol, luteolin, myricetin, naringenin, quercetin, rutin, and umbelliferone remained low and exhibited no significant differences between susceptible and resistant accessions under either control or infested conditions. Although isolated compounds showed statistical variation in control plants (apigenin and quercetin), these differences were not maintained after infestation, and their concentration ranges overlapped extensively across all accessions. Overall, the flavonoid profiles did not demonstrate any significant differences that could be associated with thrips resistance. Quercetin and eriodictyol showed significantly higher levels in susceptible accessions under control conditions (*P* <0.05), but their VIP scores remained <1 during infestation, indicating a limited role in induced resistance. Apigenin did not show significant differences between resistant and susceptible accessions under infested conditions, with VIP scores <1, suggesting they are not primary contributors to *T. parvispinus* resistance. Similarly, umbelliferone was found in higher levels in infested conditions in IIHR 4604 (0.04 μg/g) (Table S2).

Phenolic acids showed wide quantitative variation among accessions under control conditions, with 2,4-dihydroxybenzoic acid ranging 3.43 to 9.51 ng/g, 3-hydroxybenzoic acid 52.49 to 470.01 ng/g, benzoic acid 279.51 to 724.85 ng/g, caffeic acid 3.45 to 28.30 ng/g, chlorogenic acid 55.41 to 738.75 ng/g, ellagic acid 28.96–97.44 ng/g (VIP score 1.0–1.5), ferulic acid 0.59 to 5.83 ng/g (VIP score 1.0–1.5), gallic acid 0.00–3.02 ng/g (VIP score 1.5–2.0), gentisic acid 0.48 to 1.21 ng/g, o-coumaric acid 0.00 to 4.59 ng/g, p-coumaric acid 5.39 to 36.60 ng/g (VIP score 2.0), para-hydroxybenzoic acid 13.35 to 283.40 ng/g, protocatechuic acid 0.16 to 0.45 ng/g, salicylic acid 0.00 to 32.59 ng/g, sinapic acid 1.75 to 4.53 ng/g, syringic acid 0.81 to 1.97 ng/g, trans-cinnamic acid 74.45–316.11 ng/g, and vanillic acid 4.21 to 41.96 ng/g (VIP score 1.0–1.5) (Table S3, Fig. S3).

Following infestation, phenolic concentrations shifted across accessions but remained broadly comparable between resistance categories. The ranges were 4.64 to 25.80 ng/g for 2,4-dihydroxybenzoic acid, 168.18 to 670.76 ng/g for 3-hydroxybenzoic acid, 156.76 to 1558.29 ng/g (VIP score 2.0) for benzoic acid, 0.93 to 64.17 ng/g for caffeic acid, 22.05 to 5191.26 ng/g (VIP score 1.5–2.0) for chlorogenic acid, 42.36–86.85 ng/g for ellagic acid, 0.47 to 2.31 ng/g (VIP score 1.0–1.5) for ferulic acid, 0.78–36.85 ng/g (VIP score 1.0–1.5) for gallic acid, 0.67–4.73 ng/g (VIP score 1.0–1.5) for gentisic acid, 0.79 to 3.16 ng/g (VIP score 1.0 to 1.5) for o-coumaric acid, 2.97 to 31.49 ng/g (VIP score 1.0–1.5) for p-coumaric acid, 22.93 to 549.64 ng/g for para-hydroxybenzoic acid, 0.23 to 0.50 ng/g for protocatechuic acid, 0.00 to 77.69 ng/g for salicylic acid, 1.51 to 13.72 ng/g for sinapic acid, 1.33 to 1.92 ng/g for syringic acid, 175.24 to 457.37 ng/g for trans-cinnamic acid, and 5.49 to 100.99 ng/g for vanillic acid (Table S3, Fig. S3).

The phenolic acid profiling revealed distinct variations between resistant (IIHR 4550, IIHR-B-HP-79) and susceptible (IIHR 3455, IIHR 4604) accessions under control and infested conditions, supported by their respective VIP scores. Under infested conditions, 2,4-dihydroxybenzoic acid in resistant accessions [IIHR 4550: (7.66 ng/g), IIHR-B-HP-79 (25.80 ng/g)] exhibited significantly higher levels but with VIP < 1 (Table S3, Fig. S3). 3-Hydroxybenzoic acid showed higher levels in resistant accessions under both control (IIHR 4550: 56.55 ng/g, IIHR-B-HP-79: 470.01 ng/g) and infested (IIHR 4550, 653.51 ng/g; IIHR-B-HP-79, 670.76 ng/g) conditions but VIP < 1 (Table S3). Benzoic acid increased in resistant accession IIHR 4550 during infestation (1362.87 ng/g) with a VIP score > 1 under infested conditions and Caffeic Acid levels were also high in IIHR 4550 (64.17 ng/g) under infested conditions (Table S3, Fig. 4). The compounds with high VIP score like Gentisic acid (VIP = 1.5) and O-Coumaric acid (VIP = 1–1.5) (Fig. 4) were also found to be present high in both resistant and susceptible accessions.

### GC–MS profiling of epicuticular wax and leaf volatiles

**Figure 2:**
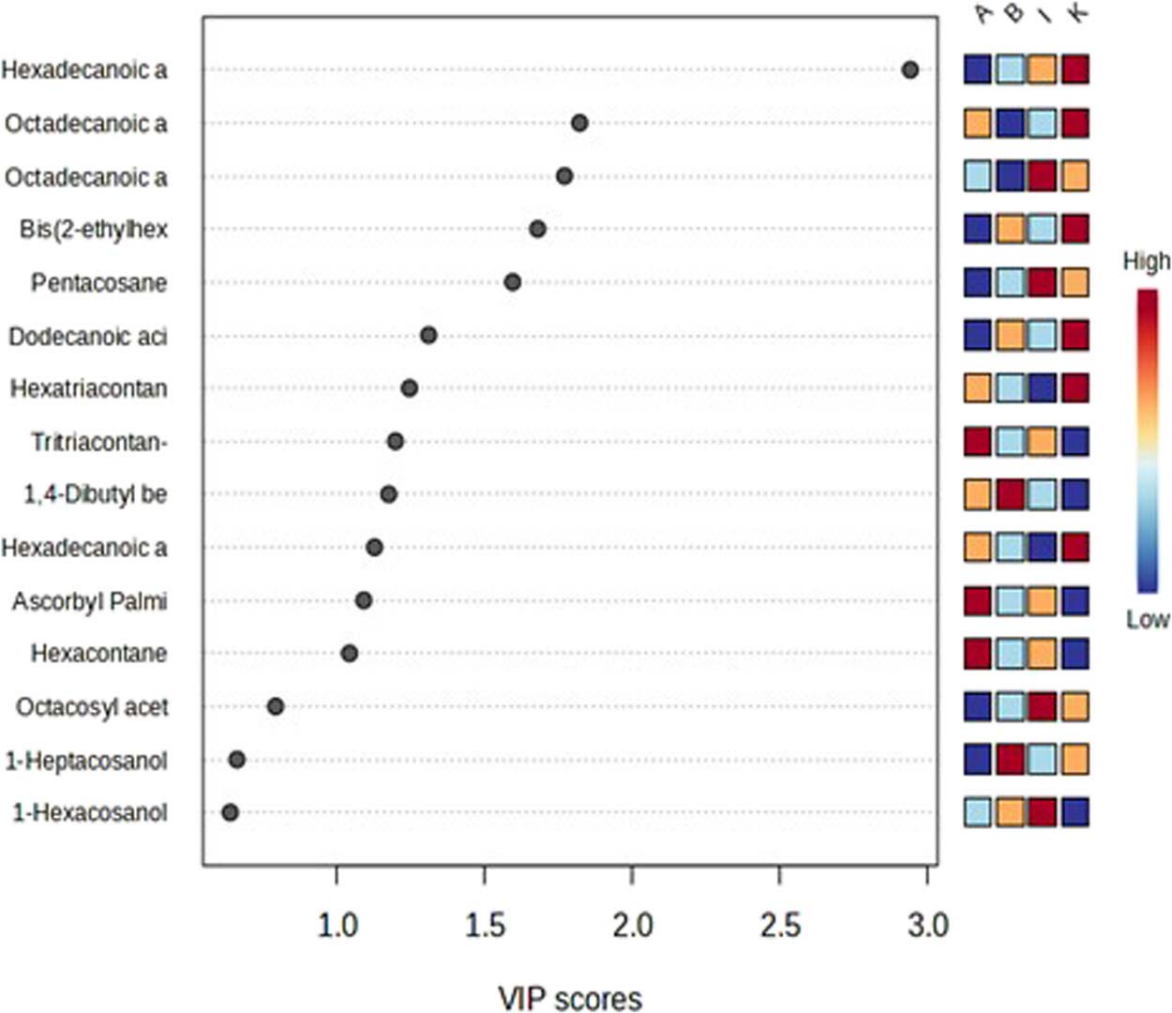
Variable Importance in Projection (VIP) scores of epicuticular wax contributing to group discrimination among chilli accessions. The heatmap indicates relative abundance across four chilli accessions: Resistant—A (IIHR 4550); I (IIHR-B-HP-79), Susceptible—K (IIHR 3455), B (IIHR 4604). Compounds highly accumulated in both resistant accessions (I and K) are suggested to play a potential role in thrips resistance. Results of 5-fold cross-validation are also shown, with the predictive performance (*R*^2^ = 0.98, *Q*^2^ = 0.75, accuracy = 0.97).

Cuticular thickness through SEM analysis revealed both compositional and structural differences in the resistant and susceptible chilli accessions. Metabolic profiling revealed that specific compounds, such as ascorbyl palmitate, tritriacontan-one, and hexacontane, with VIP scores >1 were uniquely detected in the resistant accession IIHR 4550 but were absent in other accessions including another resistant accession IIHR-B-HP-79 (Fig. 2). Conversely, eicosanoic acid, 2-[(1-oxohexadecyl)oxy]-1-[[(1-oxohexadecyl)oxy]methyl]ethyl ester and heptadecyl alcohol were exclusive to IIHR-B-HP-79, suggesting genotype-specific wax profiles (Table S7). Notably, none of these compounds were commonly abundant in both resistant accessions, implying they may not be universally reliable markers for resistance selection. However, they likely contribute to resistance in a genotype-dependent manner by reinforcing the wax barrier.

**Figure 3:**
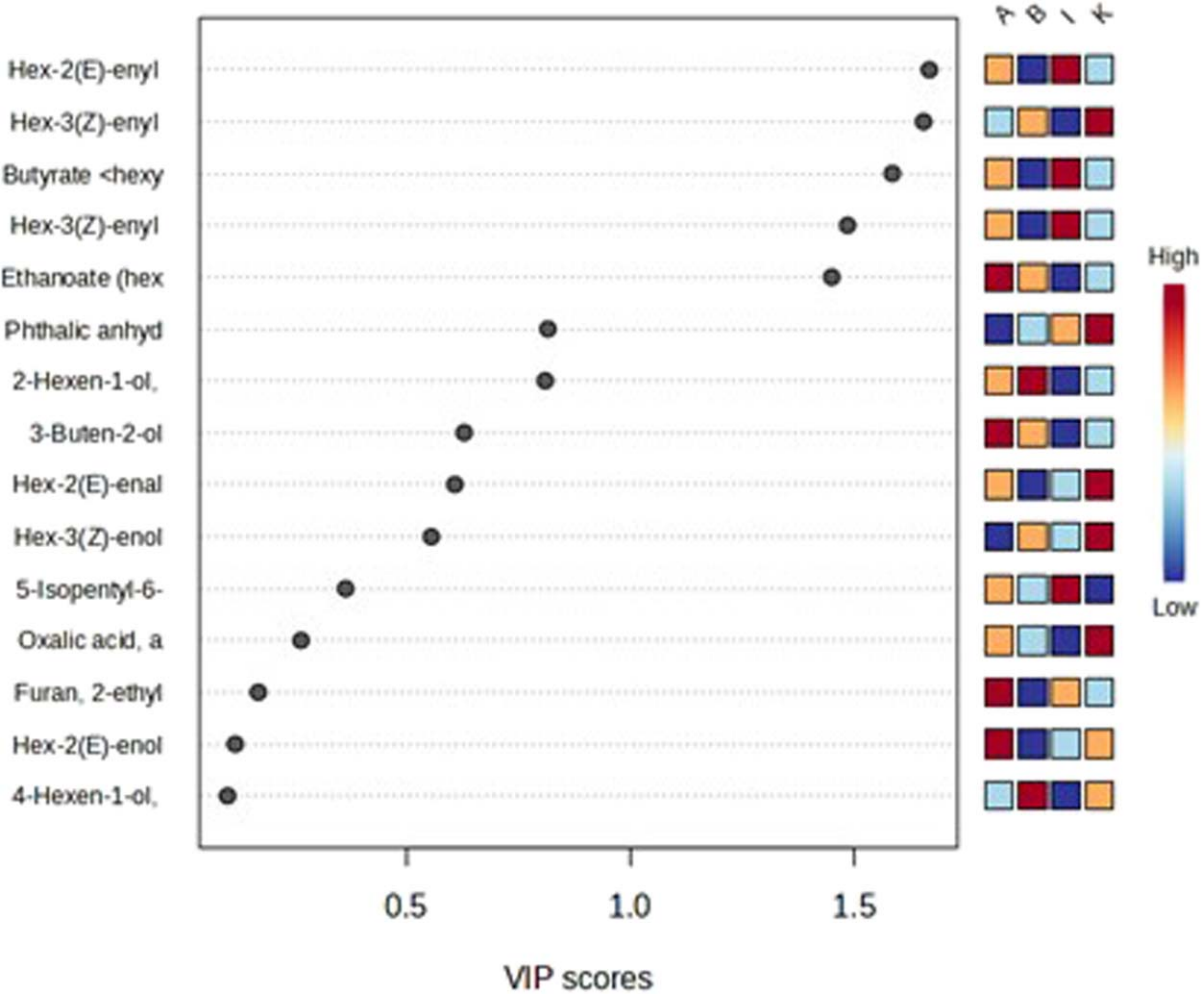
Variable Importance in Projection (VIP) scores of leaf volatiles contributing to group discrimination among chilli accessions. The heatmap indicates relative abundance across four chilli accessions: Resistant—A (IIHR 4550); I (IIHR-B-HP-79), Susceptible—K (IIHR 3455), B (IIHR 4604). Results of 5-fold cross-validation are also shown, with the predictive performance (*R*^2^ = 0.50, *Q*^2^ = 0.80, accuracy = 0.70).

**Figure 4:**
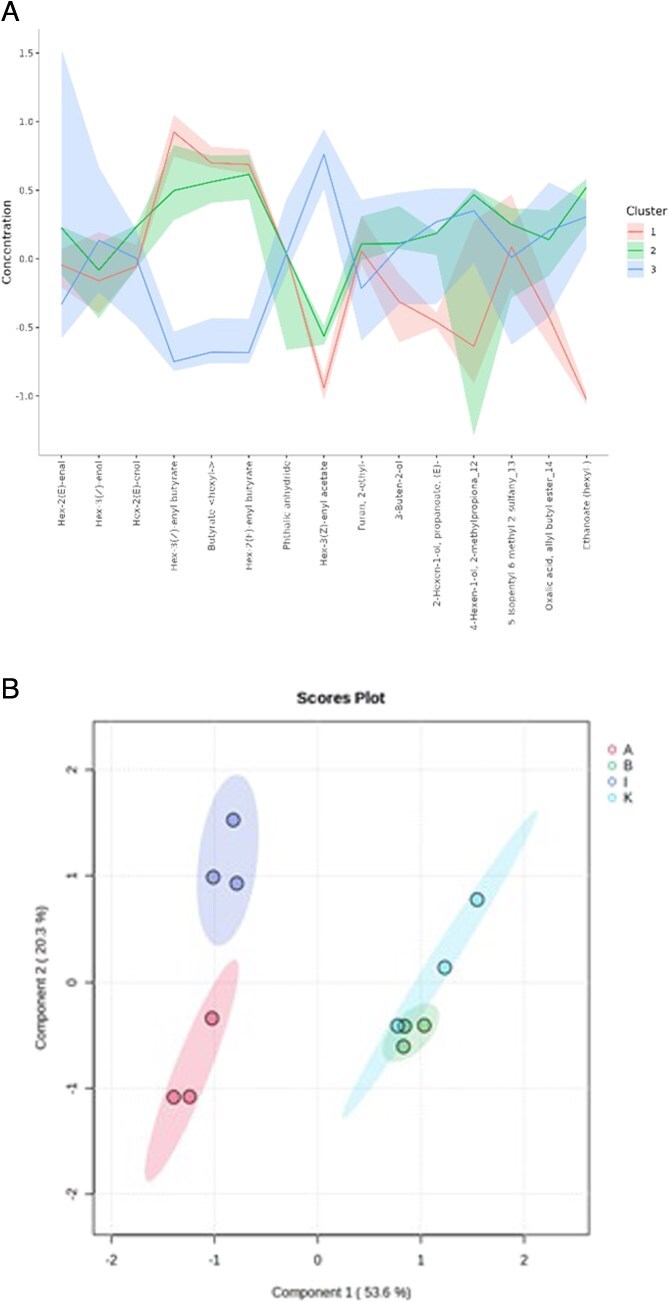
(a) Left panel shows the K-means cluster analysis. The x-axis shows variable indices and y-axis shows are relative intensities. (b) Right panel indicates the Partial Least Square Discriminant Analysis (PLS-DA) score plot for leaf volatile profiling (*F*-value: 28.206; *R*^2^: 0.91363; *P* value (based on 999 permutations): .001). Resistant—A (IIHR 4550); I (IIHR-B-HP-79), Susceptible—K (IIHR 3455), B (IIHR 4604).

In the leaf volatile profiling, certain compounds like Hex-3(Z)-enyl butyrate, Hex-2(E) enyl butyrate and butyrate hexyl were present in both resistant accessions IIHR 4550 and IIHR-B-HP-79 in significant amounts in their peak values with VIP scores >1.5 (Table S8, Fig. 3). K-means clustering analysis grouped the metabolite profiles into three distinct clusters (Fig. 4a). The clusters indicate that susceptible accessions IIHR 3455 and IIHR 4604 were grouped together in Cluster 1, while resistant accessions were divided into Cluster 2 (IIHR-B-HP-79) and Cluster 3 (IIHR 4550). This clustering suggests a clear biochemical distinction between susceptible and resistant genotypes, reinforcing the metabolic basis of thrips resistance. The PLS-DA scores plot (Fig. 4b) demonstrated a clear separation of *Capsicum* accessions based on their leaf volatile profiling. Resistant accessions, *C. chinense* (A—IIHR 4550) and *C. frutescens* (I—IIHR-B-HP-79) clustered distinctly on the negative side of Component 1, while susceptible *C. annuum* (B—IIHR 4604 and K—IIHR 3455) grouped closely on the positive side, indicating metabolic differences between resistance and susceptibility. IIHR4550 (*C. chinense* (A)) displayed greater variability in its volatile profile compared to IIHR-B-HP-79; *C. frutescens* (I), reflecting species-specific chemical contributions to resistance. Conversely, the clustering of susceptible accessions suggests a shared metabolic profile that may be linked to susceptibility. Component 1 (53.6% variance) effectively discriminated resistance traits, while Component 2 (20.3%) captured species level differences.

### Leaf dip bioassay and validation through four-arm olfactometer

Based on the results from leaf volatile profiling, commercially available Hex-3(Z)-enyl butyrate and Butyrate hexyl were used to test the effect of these compounds on *T. parvispinus*. To confirm the effect of these compounds on *T. parvispinus*, leaf discs bioassay was conducted by dipping the leaves of *C. annuum* (susceptible) in two synthetic compounds (Hex-3(Z)-enyl butyrate and Butyrate Hexyl) separately at 0.5, 1, 2, 4, 8 and 16 μl^-l^ concentrations (Table 2). Four-arm olfactometer tests were performed to validate the bioassay results. In Hex-3(Z)-enyl butyrate dipped leaves, the mean scraping percentage was low compared to control samples across concentrations. In 8 μl^-l^, treated samples showed a significant reduction in mean scraping % (24.6% ± 0.38), compared to the control (55.58% ± 5.59). Similarly, in 16 μl^-l^, the treated samples exhibited low mean scraping % (24.64 ± 0.38), which was significantly lower than the control (63.76% ± 4.15). However, no significant differences were observed at lower concentrations (0.5, 1, 2 and 4 μl^-l^). For Butyrate hexyl treatment, similar trends were observed, where higher concentrations demonstrated significant reductions in mean scraping % for treated samples. At 16 μl^-l^, treated samples had a mean scraping % of (28.66% ± 3.23) which was significantly lower than the control (38.47% ± 7.83). Lower concentrations (0.5, 1, 2, 4 and 8 μl^-l^) did not exhibit significant differences between treated and control samples.

**Table 2:** Leaf dip bioassay of two biochemical compounds against *T. parvispinus* in susceptible chilli accession

**Treatment**	**Hex-3(Z)-enyl butyrate**		**Butyrate hexyl**	
	**Control**	**Treated**	**Control**	**Treated**
	**Mean ± SE**	**Mean ± SE**	**Mean ± SE**	**Mean ± SE**
0.5 µL^-L^	69.76 ± 1.25	60.63 ± 8.88^a^	69.88 ± 3.42	69.76 ± 1.25^a^
	(0.99 ± 0.01)	(0.90 ± 0.09)	(0.99 ± 0.04)	(0.99 ± 0.01)
1 µL^-L^	57.39 ± 7.73	60.53 ± 6.43^a^	62.39 ± 7.66	57.39 ± 7.73^a^ (0.86 ± 0.08)
	(0.86 ± 0.08)	(0.89 ± 0.07)	(0.91 ± 0.08)	
2 µL^-L^	60.55 ± 4.68	53.55 ± 5.04^a^	54.49 ± 11.27	60.55 ± 4.68^a^
	(0.89 ± 0.05)	(0.82 ± 0.05)	(0.83 ± 0.12)	(0.89 ± 0.05)
4 µL^-L^	45.03 ± 5.59	62.65 ± 1.62^a^	37.81 ± 4.72	45.03 ± 4.74^a^ (0.74 ± 0.05)
	(0.74 ± 0.05)	(0.91 ± 0.02)	(0.66 ± 0.05)	
8 µL^-L^	55.58 ± 5.59	24.6 ± 0.38^b^	47.91 ± 10.16	52.24 ± 8.85^a^
	(0.84 ± 0.06)	(0.51 ± 0.04)	(0.76 ± 0.10)	(0.81 ± 0.09)
16 µL^-L^	63.76 ± 4.15	24.64 ± 0.38^b^	38.47 ± 7.83	28.66 ± 3.23^b^
	(0.93 ± 0.04)	(0.52 ± 0.02)	(0.67 ± 0.08)	(0.67 ± 0.03)
*P* value	NS	<.05	NS	<.05

Values followed by the same letter(s) within a column are not significantly different according to Duncan's Multiple Range Test (DMRT) at *P* ≤ 0.05.

**Figure 5:**
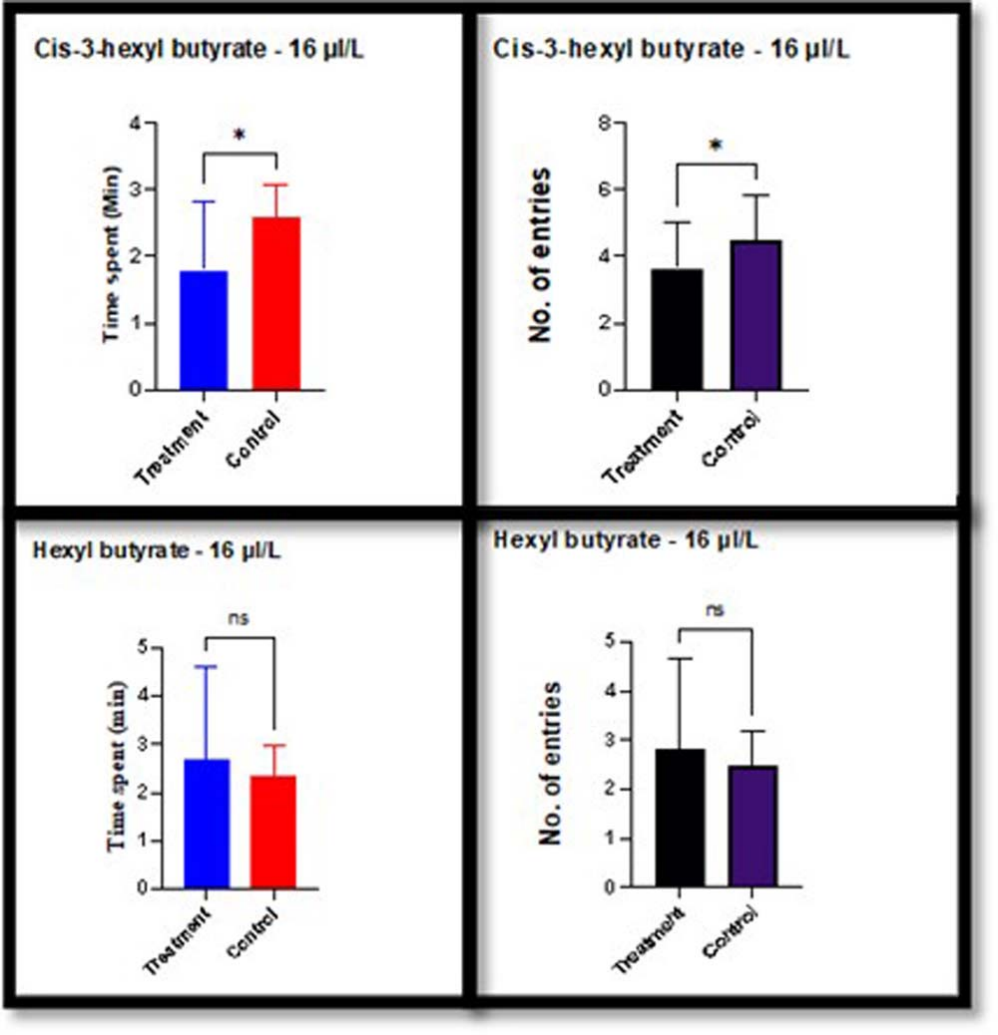
Behavioural responses of *T. parvispinus* to chilli volatiles (Hex-3(Z)-enyl butyrate and Butyrate hexyl) showing mean time spent in treatment and control arms, and mean number of entries into treatment and control arms at 16 µL^-L^ concentration. Error bars = standard error of mean; * differences significant by paired *t*-test, df = 19; df—degrees of freedom; *n* = 20.

Overall, the results indicated that the synthetic compounds, at higher concentrations, effectively validated the role of these leaf volatiles in mediating resistance responses in the bioassay. These findings emphasize the potential use of these compounds in pest resistance strategies. To further validate the findings from the leaf dip bioassay, a four-arm experiment was conducted using the two synthetic compounds, Hex-3(Z)-enyl butyrate and Butyrate hexyl, at a concentration of 16 µL^-L^(Fig. 5). The number of entries and time spent were recorded as measures of insect response. For Hex-3(Z)-enyl butyrate, a significant reduction (*P < .05*) in the number of entries was observed in the treated samples compared to the control, as evident in both replicates. However, at 8 µL^-L^, the differences were found to be non-significant, further supporting the concentration-dependent response observed in the bioassay.

In contrast, for Butyrate hexyl, no significant differences (*P >* 0.05) were observed between the treated and control groups for both the number of entries and the time spent. These results indicate that Butyrate hexyl was less effective in influencing insect behaviour compared to Hex-3(Z)-enyl butyrate, aligning with the bioassay findings. These results corroborate the outcomes of the leaf dip bioassay, further validating the role of Hex-3(Z)-enyl butyrate as a potential volatile compound mediating *T. parvispinus* resistance.

## Discussion

The present study delved into the biochemical and structural basis of *T. parvispinus* resistance in chilli by examining leaf trichome types and epicuticular wax studies followed by profiling of sugars, flavonoids and phenolic acids in resistant (IIHR 4550 and IIHR-B-HP-79) and susceptible (IIHR 3455 and IIHR 4604) accessions under control and infested conditions. The trichome count and type on the abaxial surface of resistant (R) and susceptible (S) accessions have shown no significant differences (Table 1). Resistant accessions IIHR 4550 and IIHR-B-HP-79, displayed higher trichome counts (60.00 ± 4.59 and 37.60 ± 4.93, respectively) compared to the susceptible accessions IIHR 3455 and IIHR 4604, which exhibited lower counts (12.80 ± 2.76 and 26.20 ± 4.33, respectively). Previous studies have also reported the presence of non-glandular trichomes in *C. annuum*, *C. chinense*, and *C. frutescens* species, highlighting their structural rather than defensive role in pest resistance [25, 26]. There were no significant differences between IIHR-B-HP-79, IIHR 3455 and IIHR 4604. Instead, the observed resistance may be attributed to other factors such as higher densities of non-glandular trichomes, elevated levels of defensive metabolites, or underlying genetic resistance mechanisms. Similar cases where resistance occurred in the absence of glandular trichomes have also been reported [27], indicating that the role of trichome type in pest resistance may be pest-specific or vary with accession background.

Profiling results of sugars showed sucrose as a signalling molecule and energy source, as resistant accessions exhibited a significant increase in its levels under infested conditions (IIHR 4550, 0.93 μg/g; IIHR-B-HP-79, 2.75 μg/g) suggesting sucrose involvement in energy mobilization and signalling pathways that activate defence responses during pest attacks. Sucrose may contribute to stress responses by acting as an energy source for induced defences or participating in signalling pathways. Sucrose's role in herbivore resistance has been supported by studies highlighting its involvement in metabolic adjustments and activation of phenylpropanoid pathway during stress [20, 28, 29].

Furthermore, sucrose contributes to the regulation of ROS signaling, which plays a key role in defense gene activation and hypersensitive responses [30]. In contrast, inositol, despite its high concentration in susceptible accessions, appears to function as a secondary metabolite as its concentration was low/ negligible in resistant accessions involved in pest induced stress response rather than a resistance marker. Sugars such as arabinose, fucose and rhamnose displayed significant variations but had VIP scores <1 under infestation, suggesting limited relevance in induced resistance. This pattern reinforces the hypothesis that these sugars contribute to basal metabolic processes or structural reinforcement rather than direct defence against pest [12]. Several flavonoids including catechin, quercetin, kaempferol and umbelliferone showed differential accumulation across chilli accessions, with some exhibiting higher levels and VIP > 1 in resistant genotypes following infestation. However, no single flavonoid was consistently present in both IIHR 4550 and IIHR-B-HP-79, suggesting a role in inducible rather than constitutive resistance. While their direct contribution to *T. parvispinus* resistance appears genotype-specific, flavonoids may act synergistically with phenolic acids through the phenylpropanoid pathway to enhance defense responses [31]. Flavonoids may still play indirect roles in plant defence through cross-talk with other metabolic pathways. Studies have shown that some flavonoids participate in the regulation of reactive oxygen species (ROS) or in signalling networks [32, 33]. Their functions as feeding deterrents, enzyme inhibitors, and stress signal mediators support their relevance in a broader metabolic resistance network [12, 34]. Phenolic acids demonstrated significant potential in resistance, with certain compounds showing pronounced differences in resistant accessions. Benzoic acid (IIHR 4550: 1362.87 ng/g; VIP > 1) and caffeic acid (IIHR 4550: 64.17 ng/g) were elevated under infestation. Phenolic acids have been reported to enhance cell wall rigidity and scavenge reactive oxygen species during stress. Some phenolic acids increased in one resistant line but remained unchanged or even decreased in the other. This variability suggests that phenolic acids may be induced as part of a localized defence response rather than representing a stable, constitutive resistance trait. Therefore, phenolic acids although involved in stress responses, were not conclusively linked to inherent *T. parvispinus* resistance across the studied accessions. However, phenolic acids play a very important role in defence mechanism against insect resistance and their contribution to oxidative stress mitigation and cell wall strengthening [12, 35] was well studied. Compounds like 3-hydroxybenzoic acid and 2,4-dihydroxybenzoic acid, despite lower VIP scores, were consistently higher in resistant accessions, implying their auxiliary role in resistance pathways. Phenolic acids have also been linked to insect resistance by contributing to the inhibition of digestive enzymes in herbivores [12]. The significant presence of these metabolites emphasizes the complexity of phenolic acid-mediated defence mechanisms [35]. Although sugars, flavonoids and phenolic acids are known to play important roles in plant defense, either individually or through synergistic interactions via pathways, our study did not detect consistently elevated or suppressed levels of these compounds in resistant accessions under thrips-infested conditions. This suggests that their contribution to resistance may be genotype-specific and possibly driven by complex interactions among these metabolites rather than their individual abundance. It is plausible that a coordinated or synergistic effect among these secondary metabolites, even at moderate concentrations, may enhance resistance in certain genotypes through pathway cross-talk or regulatory modulation.

To understand the resistant mechanism involving the physical barrier and repulsion of pests in resistant accessions profiling of epicuticular wax and leaf volatiles were performed. Metabolites with VIP scores greater than 1 may serve as potential biomarkers for the plant’s response to pest infection [36]. Due to the complexity of the GC–MS profiles obtained from resistant and susceptible accessions, only compounds with a peak area greater than 1% in at least one accession (Table S9) were selected for further analysis using MetaboAnalyst 6.0. Multivariate analysis was performed to understand the dimension and importance of the compounds. Epicuticular wax profiling and SEM analysis revealed both structural and compositional differences among chilli accessions. Ascorbyl Palmitate, Tritriacontan-one and Hexacontane were detected exclusively in IIHR 4550, whereas Pentacosane and Octadecanoic acid derivatives were unique to IIHR-B-HP-79. These compounds had VIP scores >1 but were not common to both resistant genotypes, indicating genotype-specific roles in resistance. Samuels *et al.* [16] and Eigenbrode and Espelie [13] similarly emphasized that both the physical structure and chemical composition of epicuticular waxes play vital roles in mediating insect-plant interactions. In the present study, IIHR 4550 likely relies more on chemical deterrence via wax composition, while IIHR-B-HP-79 may depend on its thicker physical barrier, reinforcing the idea that multiple, non-overlapping mechanisms may underlie thrips resistance in different chilli genotypes.

Plant volatiles can deter insects by altering their feeding behaviour and survival [37]. The PLS-DA scores plot revealed distinct clustering between *C. chinense* (IIHR 4550) and *C. frutescens* (IIHR-B-HP-79), indicating species-specific volatile profiles. These findings align with reports highlighting the role of volatiles in insect resistance, where resistant genotypes produce deterrent compounds that are absent in susceptible accessions [38]. Hex-3(Z)-enyl butyrate was detected in high concentrations in both accessions, suggesting that its production is not strictly species-specific, but may be associated with resistance-related metabolic pathways. Notably, in the four-arm olfactometer assay, this compound elicited a significant repellency response in *T. parvispinus*, further supporting its potential role in behavioural resistance. These findings indicate that Hex-3(Z)-enyl butyrate may contribute to resistance across accessions, regardless of species classification. Interestingly, while Butyrate hexyl showed lower impact compared to Hex-3(Z)-enyl butyrate in four-arm olfactometer studies implies the specificity of volatile-mediated defence mechanisms. The concentration-dependent response observed in the bioassays further validates these compounds roles as key mediators of *T. parvispinus* resistance. Volatile organic compounds are known to serve dual roles as direct deterrents and signals that recruit natural enemies of herbivores [39], making them valuable components of integrated pest management strategies.

## Conclusion

This study highlights the synergistic interaction between metabolic and structural traits in conferring *T. parvispinus* resistance in chilli. Key findings include the identification of Hex-3(Z)-enyl butyrate as a potent volatile compound and sucrose as a key metabolic marker for resistance. The validation of volatile compounds through bioassays and olfactometer tests emphasizes that this can be further explored in integrated pest management (IPM) strategies. These findings provide crucial biochemical markers for future breeding programs and suggest practical applications in pest management strategies. Further focusing on evaluating the efficacy of identified volatile compounds under field conditions for developing IPM strategies, exploring the genetic and molecular metabolic pathways of these compounds will accelerate the resistance breeding programs.

## Data analysis

Statistical analysis was conducted using Metaboanalyst 6.0 software [22] and IBM SPSS 29.0.1.0 [23] software. All the data in LC–MS, GC–MS profiling was normalized, log transformed and pareto scaled in the Metaboanalyst 6.0 software. The original mean values, p value significance, FDR ratio are presented in Tables S1–S3. The original and normalized mean values with the standard errors are provided in Tables S4–S6. The Variable Importance in Projection (VIP) score was calculated using Partial Least Squares Regression (PLSR) to identify key metabolites associated with *T. parvispinus* resistance in chilli. VIP scores were computed using a formula that incorporates the weights and scores of variables across all components of the PLSR model, with VIP > 1 indicating significant contributions to the model. This threshold highlights metabolites that are biologically and statistically relevant, as they contribute substantially to explaining the observed variation in resistance or susceptibility. For further validation studies, only those metabolites with VIP > 1 that also showed consistent and elevated expression in resistant accessions were selected, ensuring a focus on compounds most likely to be associated with resistance traits. Variables with VIP scores below 1 were considered less important and excluded from further analysis to reduce dimensionality and focus on impactful traits. This approach ensures the identification of critical metabolites for resistance breeding while eliminating noise and less relevant contributors. VIP scores are calculated from Partial Least Squares Discriminant Analysis in Metaboanalyst 6.0 software.PLS-DA score plot and K-Means clustering was calculated from Metaboanalyst 6.0 software. The IBM SPSS 29.0.1.0 was used for ANOVA and post-hoc tests. Leaf dip bioassay scraping damage % (cm2) was quantified using Image J software [24].

A leaf disc bioassay was conducted using leaves treated with volatiles and distilled water as solvent. The test concentration range (0.5–16 μl-l) was developed based on semi-quantitative GC–MS profiling of epicuticular wax/volatile extracts from resistant accessions. To evaluate dose-dependent responses, we initiated serial dilutions starting from 0.5 μl-l, creating a gradient of five concentrations (0.5, 2, 4, 8, and 16 μl-l). This approach allowed us to simulate relative biological exposure and assess behavioural effects across a range of physiologically relevant concentrations. This range was used consistently across both leaf dip bioassays and four-arm olfactometer assays to compare behavioural and contact responses of *T. parvispinus*. Treated leaves were dried and placed on 1.5% agar medium and 10 nymphs of *T. parvispinus* were released onto each disc. Each concentration was replicated thrice and scraping damage was quantified using ImageJ software [20] after 72 h. Four-arm olfactometer test was employed to validate the bioassay results and assess *T. parvispinus* behavioural responses to plant volatiles under controlled conditions (27 ± 1°C, diffused lighting, black walls). Behavioural assays were conducted following the procedure described by Jayanthi *et al.* [21]. A single adult female *T. parvispinus* was released into the central chamber of a four-arm olfactometer and allowed to acclimate for 2 min before observations were recorded for 10 min. The airflow was maintained at 350 ml/min, with one arm containing 10 μl of the plant volatile of interest applied to filter paper, while the remaining three arms received 10 μl of distilled water as solvent blanks. Distilled water was used as a neutral solvent to minimize any potential influence on thrips behaviour. The apparatus was rotated every 2 min to eliminate positional bias, and 20 replicates were performed. Time spent and the number of entries into each arm were recorded using Olfa software (OLFA, Udine, Italy). The inclusion of water-only controls ensured that the behavioural responses observed were attributable to the plant volatiles and not due to solvent effects

Leaf dip bioassay and four arm olfactometer bioassay.

## Supplementary Material

Web_Material_uhaf346

## Data Availability

All the data are available.
