# Supplementary material for: Hex-3(Z)-enyl butyrate: a key volatile compound conferring resistance against Southeast Asian Thrips (Thrips parvispinus) in Capsicum spp
Source: Hortic Res. 2026 Jan 13;13(5):uhaf346. doi: 10.1093/hr/uhaf346 (PMC13150850; doi:10.1093/hr/uhaf346)
Supplement: Web_Material_uhaf346 [file web_material_uhaf346.zip › Supplementary figures.docx]

**Supplementary Figures:**


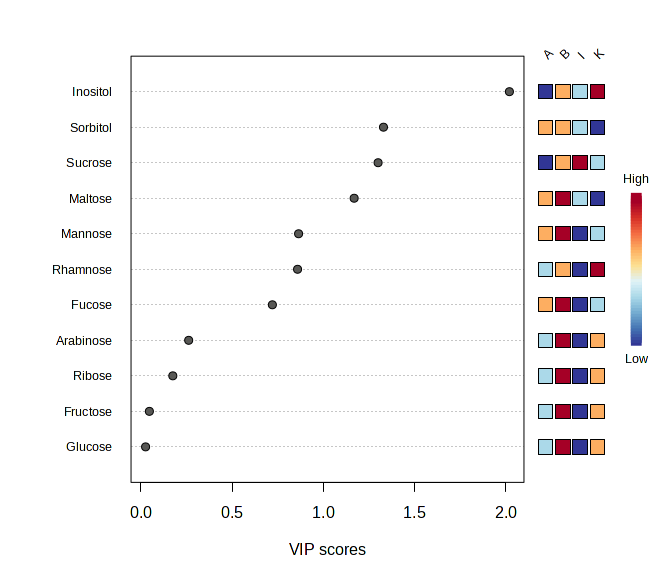

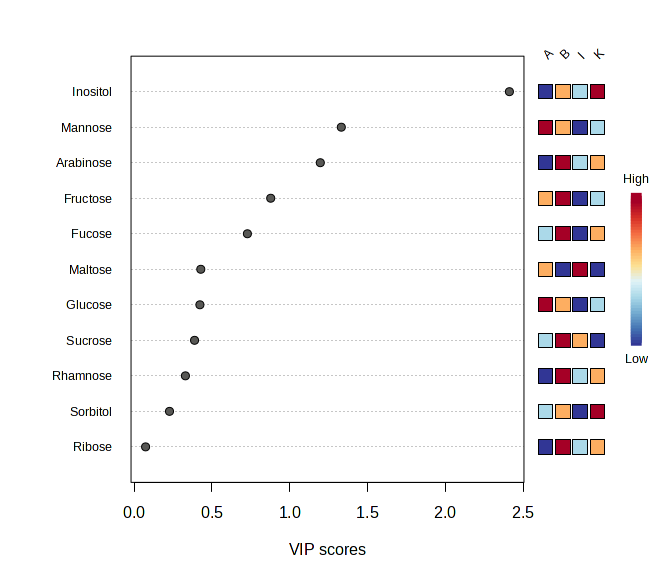


**Supplementary Figure 1: Variable Importance in Projection (VIP) scores of sugars contributing to group discrimination among chilli accessions under control (Left panel) and thrips-infested conditions (Right panel). The heatmap indicates relative abundance across four chilli accessions: Resistant - A (IIHR 4550); I (IIHR-B-HP-79), Susceptible – K (IIHR 3455), B (IIHR 4604). Results of 5-fold cross-validation are also shown, with the control model exhibiting strong predictive performance (R² = 0.99, Q² = 0.97, accuracy = 0.97), while the infested model had a high R² (0.99), Q² (0.85) and accuracy (0.60).**


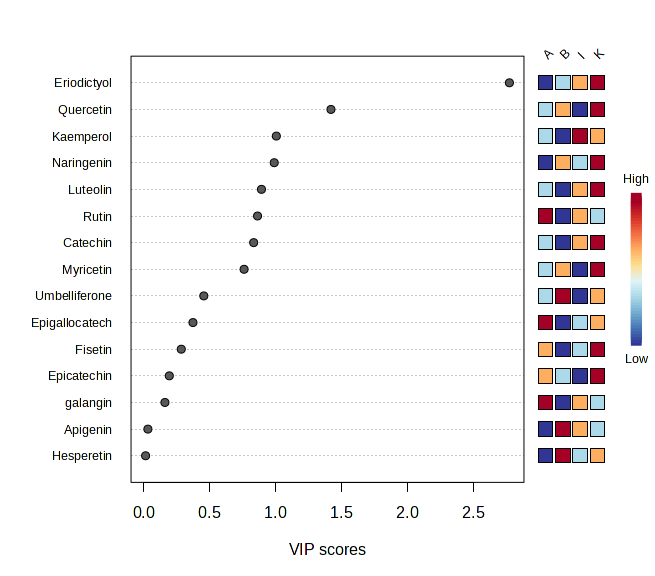

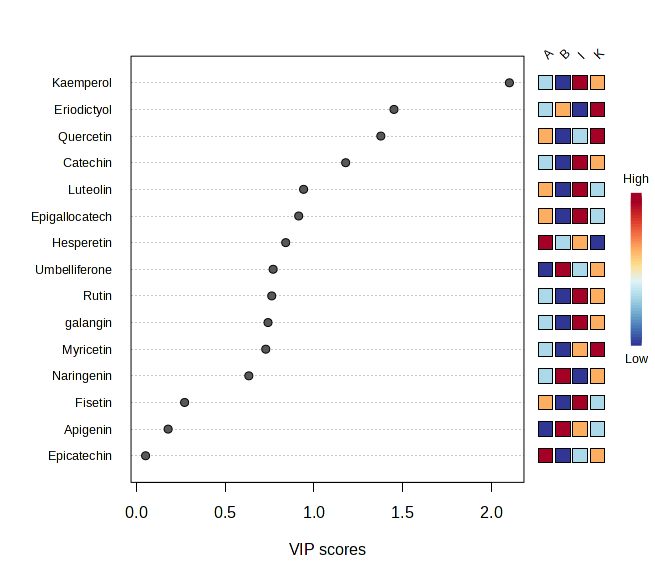


**Supplementary Figure 2: Variable Importance in Projection (VIP) scores of flavonoids contributing to group discrimination among chilli accessions under control (Left panel) and thrips-infested conditions (Right panel). The heatmap indicates relative abundance across four chilli accessions: Resistant - A (IIHR 4550); I (IIHR-B-HP-79), Susceptible – K (IIHR 3455), B (IIHR 4604). Results of 5-fold cross-validation are also shown, with the control model exhibiting predictive performance (R² = 0.99, Q² = 0.97, accuracy = 0.97), while the infested model had R² (0.99), Q² (0.00) and accuracy (0.60).**


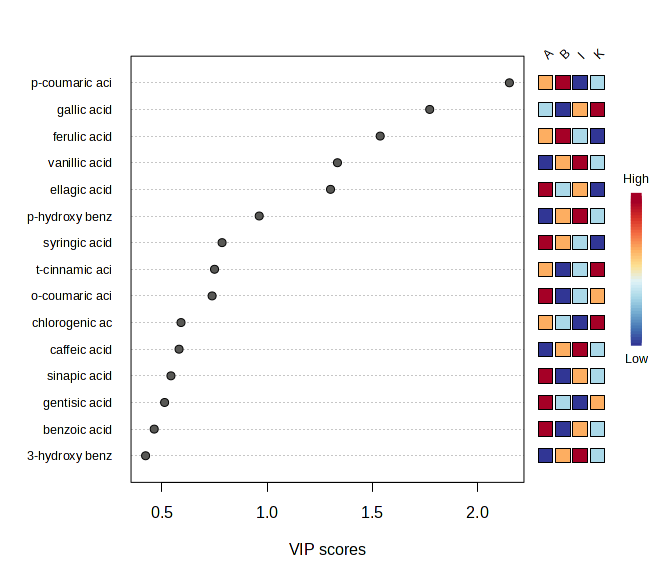

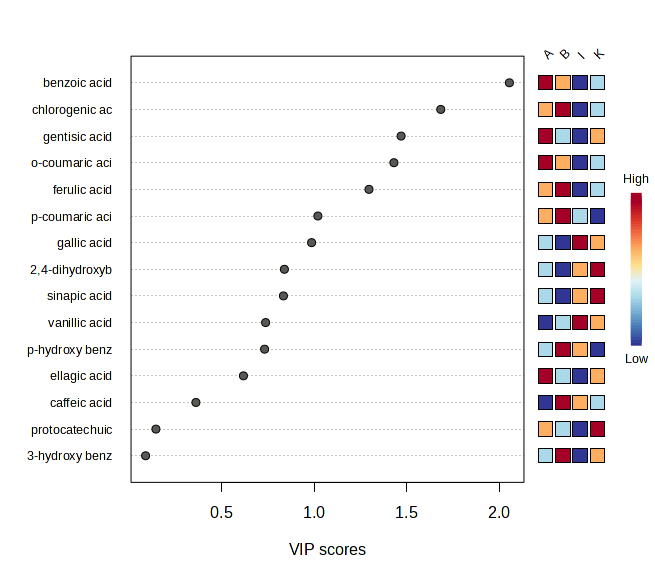


**Supplementary Figure 3: Variable Importance in Projection (VIP) scores of phenolic acids contributing to group discrimination among chilli accessions under control (Left panel) and thrips-infested conditions (Right panel). The heatmap indicates relative abundance across four chilli accessions: Resistant - A (IIHR 4550); I (IIHR-B-HP-79), Susceptible – K (IIHR 3455), B (IIHR 4604). Results of 5-fold cross-validation are also shown, with the control model exhibiting predictive performance (R² = 0.95, Q² = 0.80, accuracy = 0.97), while the infested model had R² (0.93), Q² (0.80) and accuracy (0.97).**
