# Supplementary material for: Hex-3(Z)-enyl butyrate: a key volatile compound conferring resistance against Southeast Asian Thrips (Thrips parvispinus) in Capsicum spp
Source: Hortic Res. 2026 Jan 13;13(5):uhaf346. doi: 10.1093/hr/uhaf346 (PMC13150850; doi:10.1093/hr/uhaf346)
Supplement: Web_Material_uhaf346 [file web_material_uhaf346.zip › Supplementary tables.docx]

**Supplementary Tables:**

**Supplementary Table 1 Profiling of sugars by LCMS method between two susceptible and two resistant parents**

| **Sugars** | **Mean values (µg/g) - Control** | | | | | | **Mean values (µg/g) -Infested** | | | | | | |
| --- | --- | --- | --- | --- | --- | --- | --- | --- | --- | --- | --- | --- | --- |
|  | **Susceptible** | | **Resistant** | | **P -value** | **FDR value** | **Susceptible** | | **Resistant** | | | **P -value** | **FDR value** |
|  | **IIHR 3455** | **IIHR 4604** | **IIHR 4550** | **IIHR -B-HP-79** |  |  | **IIHR 3455** | **IIHR 4604** | **IIHR 4550** | **IIHR -B-HP-79** | |  |  |
| Arabinose | 0.30 | 0.32 | 0.67 | 0.38 | *** | 0 | 0.35 | 0.27 | 0.24 | | 0.41 | NS | - |
| Fructose | 495.08 | 3596.10 | 433.62 | 144.47 | ** | 0 | 502.42 | 711.52 | 552.86 | | 162.91 | NS | - |
| Fucose | 0.14 | 0.13 | 0.55 | 0.06 | *** | 0 | 0.58 | 0.21 | 0.80 | | 0.11 | ** | 0.00 |
| Glucose | 316.44 | 135.15 | 447.76 | 158.09 | ** | 0 | 414.63 | 372.15 | 117.01 | | 294.09 | NS | - |
| Inositol | 8.74 | 0.14 | 0.10 | 0.23 | *** | 0 | 0.33 | 0.00 | 0.09 | | 0.00 | *** | 0.00 |
| Maltose | 4.84 | 9.19 | 63.91 | 62.77 | * | 0.04 | 1.80 | 8.42 | 1.04 | | 24.55 | NS | - |
| Mannose | 13.64 | 14.71 | 74.95 | 20.80 | *** | 0 | 3.39 | 15.55 | 2.39 | | 3.53 | NS | - |
| Rhamnose | 0.47 | 0.03 | 0.15 | 0.04 | *** | 0 | 0.20 | 0.12 | 0.66 | | 0.12 | *** | 0.00 |
| Ribose | 36.07 | 40.71 | 75.43 | 45.20 | * | 0.02 | 28.01 | 33.37 | 197.70 | | 39.21 | NS | - |
| Sorbitol | 1.16 | 1.96 | 14.45 | 5.15 | *** | 0 | 5.20 | 7.69 | 2.73 | | 3.95 | NS | - |
| Sucrose | 0.17 | 0.34 | 0.14 | 0.29 | *** | 0 | 0.29 | 0.88 | 0.93 | | 2.75 | ** | 0.02 |
| Xylose | 0 | 0 | 0 | 0 | NS | - | 0 | 0 | 0 | | 0 | NS | - |

(*- Significance at 0.05 level; **- Significance at 0.01 level; *** - Significance at 0.001 level; Peak area values are log transformed; FDR- False Discovery Rate; P- values are based on ANOVA)

**Supplementary Table 2 Profiling of flavonoids by LCMS method between two susceptible and two resistant parents**

| **Flavonoids** | **Mean values ( µg/g) - Control** | | | | | | **Mean values ( µg/g) -Infested** | | | | | |
| --- | --- | --- | --- | --- | --- | --- | --- | --- | --- | --- | --- | --- |
|  | **Susceptible** | | **Resistant** | | **P -value** | **FDR value** | **Susceptible** | | **Resistant** | | **P -value** | **FDR value** |
|  | **IIHR 3455** | **IIHR 4604** | **IIHR 4550** | **IIHR -B-HP-79** |  |  | **IIHR 3455** | **IIHR 4604** | **IIHR 4550** | **IIHR -B-HP-79** |  |  |
| Apigenin | 0.14 | 0.20 | 0.30 | 0.85 | ** | 0.01 | 0.21 | 0.85 | 0.16 | 0.23 | NS | - |
| Catechin | 3.71 | 3.75 | 1.14 | 2.68 | NS | - | 2.32 | 0.85 | 2.07 | 14.87 | NS | - |
| Epicatechin | 4.87 | 4.13 | 3.40 | 2.30 | NS | - | 4.04 | 4.15 | 4.50 | 2.57 | NS | - |
| Epigallocatechin | 1.94 | 2.03 | 1.90 | 1.32 | NS | - | 1.28 | 1.24 | 2.20 | 1.85 | NS | - |
| Eriodictyol | 0.02 | 0.00 | 0.00 | 0.01 | ** | 0.02 | 0.00 | 0.00 | 0.00 | 0.00 | NS | - |
| Fisetin | 0.00 | 0.00 | 0.00 | 0.00 | NS | - | 0.00 | 0.00 | 0.00 | 0.00 | NS | - |
| galangin | 0.02 | 0.03 | 0.03 | 0.02 | NS | - | 0.02 | 0.02 | 0.02 | 0.03 | NS | - |
| Hesperetin | 0.38 | 0.31 | 0.45 | 0.40 | NS | - | 0.29 | 0.33 | 0.43 | 0.30 | NS | - |
| Kaempferol | 0.02 | 0.03 | 0.03 | 0.05 | NS | - | 0.08 | 0.03 | 0.04 | 0.31 | NS | - |
| Luteolin | 4.59 | 3.37 | 2.27 | 3.25 | NS | - | 5.45 | 1.27 | 6.56 | 14.64 | NS | - |
| Myricetin | 6.15 | 3.50 | 5.13 | 2.65 | NS | - | 4.78 | 3.45 | 4.61 | 3.08 | NS | - |
| Naringenin | 1.38 | 0.97 | 0.99 | 1.42 | NS | - | 1.16 | 0.98 | 0.89 | 0.70 | NS | - |
| Quercetin | 3.57 | 1.61 | 1.96 | 1.76 | *** | 0.01 | 3.03 | 1.27 | 2.05 | 1.32 | NS | - |
| Rutin | 0.67 | 2.72 | 0.91 | 1.38 | NS | - | 1.90 | 1.06 | 1.93 | 1.82 | NS | - |
| Umbelliferone | 0.01 | 0.02 | 0.02 | 0.02 | NS | - | 0.01 | 0.04 | 0.01 | 0.01 | *** | 0.01 |

(*- Significance at 0.05 level; **- Significance at 0.01 level; *** - Significance at 0.001 level; Peak area values are log transformed; FDR- False Discovery Rate; P- values are based on ANOVA)

**Supplementary Table 3 Profiling of phenolic acids by LCMS method between two susceptible and two resistant parents**

| **Phenolic acids** | **Mean values (ng/g) - Control** | | | | | | **Mean values ( ng/g) -Infested** | | | | | |
| --- | --- | --- | --- | --- | --- | --- | --- | --- | --- | --- | --- | --- |
|  | **Susceptible** | | **Resistant** | | **P -value** | **FDR value** | **Susceptible** | | **Resistant** | | **P -value** | **FDR value** |
|  | **IIHR 3455** | **IIHR 4604** | **IIHR 4550** | **IIHR -B-HP-79** |  |  | **IIHR 3455** | **IIHR 4604** | **IIHR 4550** | **IIHR -B-HP-79** |  |  |
| 2,4-Dihydroxybenzoic Acid | 9.51 | 5.04 | 8.26 | 3.43 | * | 0.004 | 7.05 | 4.64 | 7.66 | 25.80 | * | 0.000 |
| 3-Hydroxy Benzoic Acid | 52.49 | 178.85 | 56.55 | 470.01 | ** | 0.000 | 189.75 | 168.18 | 653.51 | 670.76 | ** | 0.000 |
| Benzoic Acid | 279.51 | 278.69 | 538.69 | 724.85 | ** | 0.000 | 156.76 | 1558.29 | 1362.87 | 926.14 | ** | 0.002 |
| Caffeic Acid | 4.24 | 17.85 | 3.45 | 28.30 | ** | 0.000 | 1.22 | 0.93 | 64.17 | 12.99 | ** | 0.000 |
| Chlorogenic Acid | 738.75 | 695.67 | 639.54 | 55.41 | *** | 0.000 | 361.59 | 1691.61 | 5191.26 | 22.05 | *** | 0.000 |
| Ellagic Acid | 28.96 | 59.31 | 74.14 | 97.44 | NS | - | 48.72 | 86.85 | 42.36 | 45.18 | NS | - |
| Ferulic Acid | 0.59 | 5.83 | 1.45 | 2.78 | ** | 0.000 | 0.47 | 1.37 | 2.31 | 1.25 | ** | 0.000 |
| Gallic Acid | 1.64 | 0.00 | 0.78 | 3.02 | *** | 0.000 | 1.01 | 0.78 | 0.78 | 36.85 | *** | 0.000 |
| Gentisic Acid | 0.90 | 1.21 | 1.07 | 0.48 | * | 0.003 | 0.78 | 4.73 | 1.19 | 0.67 | * | 0.003 |
| O-Coumaric Acid | 0.93 | 0.00 | 4.59 | 0.91 | *** | 0.014 | 0.79 | 3.16 | 2.02 | 1.11 | *** | 0.000 |
| P-Coumaric Acid | 5.39 | 36.60 | 19.86 | 7.45 | *** | 0.000 | 2.97 | 7.20 | 12.85 | 31.49 | **** | 0.000 |
| Para-Hydroxy Benzoic Acid | 20.54 | 97.55 | 13.35 | 283.40 | *** | 0.000 | 22.93 | 51.75 | 290.35 | 549.64 | *** | 0.000 |
| Protocatechuic Acid | 0.45 | 0.42 | 0.29 | 0.16 | ** | 0.001 | 0.40 | 0.31 | 0.50 | 0.23 | ** | 0.001 |
| Salicylic Acid | 9.03 | 0.00 | 32.59 | 21.59 | *** | 0.00 | 33.11 | 71.56 | 0.00 | 77.69 | *** | 0.000 |
| Sinapic Acid | 1.76 | 1.75 | 3.32 | 4.53 | NS | - | 3.38 | 2.42 | 1.51 | 13.72 | NS | - |
| Syringic Acid | 0.81 | 1.97 | 1.13 | 1.92 | NS | - | 1.87 | 1.58 | 1.33 | 1.92 | NS | - |
| Trans-Cinnamic Acid | 175.31 | 74.45 | 169.51 | 316.11 | ** | 0.00 | 179.05 | 175.24 | 284.42 | 457.37 | ** | 0.000 |
| Vanillic Acid | 7.49 | 19.75 | 4.21 | 41.96 | *** | 0.00 | 7.13 | 5.49 | 10.05 | 100.99 | *** | 0.000 |

(*- Significance at 0.05 level; **- Significance at 0.01 level; *** - Significance at 0.001 level; Peak area values are log transformed; FDR- False Discovery Rate; P- values are based on ANOVA)

**Supplementary Table 4: Profiling of sugars with original mean value concentrations (µg/g)** ±**standard error values and normalized mean values**± **standard error values by LCMS method between two susceptible and two resistant parents**

| **Sugars** | **Mean values (µg/g) - Control** | | | | **Mean values (µg/g) -Infested** | | | |
| --- | --- | --- | --- | --- | --- | --- | --- | --- |
|  | **Susceptible** | | **Resistant** | |  | | **Resistant** | |
|  |  |  |  |  | **Susceptible** | |  |  |
|  | **IIHR 3455** | **IIHR 4604** | **IIHR 4550** | **IIHR -B-HP-79** | **IIHR 3455** | **IIHR 4604** | **IIHR 4550** | **IIHR -B-HP-79** |
| Arabinose | 0.31±0.01 (0.05±0.00) | 0.32±0.01 (0.10±0.00) | 0.67±0.03 (0.05±0.01) | 0.38±0.02 (0.03±0.01) | 0.35±0.01 (0.14±0.02) | 0.27±0.01 (0.06±0.02) | 0.24±0.01 (0.11±0.02) | 0.41±0.02 (0.09±0.01) |
|  |  |  |  |  |  |  |  |  |
| Fructose | 495.08±2.27 (2.82±0.00) | 3596.10±2649.23 (3.11±0.09) | 433.62±182.44 (2.32±0.31) | 144.47±61.81 (2.12±0.33) | 502.42±358.71 (2.91±0.15) | 711.52±7.79 (2.85±0.03) | 552.86±236.62 (2.87±0.10) | 162.91±123.32 (2.22±0.18) |
| Fucose | 0.14±0.01 (0.02±0.00) | 0.13±0.01 (0.05±0.00) | 0.55±0.01 (0.04±0.00) | 0.06±0.01 (0.01±0.00) | 0.58±0.01 (0.22±0.03) | 0.21±0.01 (0.04±0.02) | 0.80±0.01 (0.30±0.06) | 0.11±0.00 (0.02±0.00) |
| Glucose | 316.44±2.43 (2.57±0.00) | 135.15±2.43 (2.02±0.13) | 447.76±9.75 (2.63±0.20) | 158.09±67.71 (2.20±0.33) | 414.63±351.52 (2.30±0.36) | 372.15±13.23 (2.52±0.01) | 117.01±51.68 (2.07±0.14) | 294.09±178.40 (2.79±0.09) |
| Inositol | 8.74±0.25 (0.75±0.01) | 0.14±0.00 (0.05±0.00) | 0.10±0.00 (0.01±0.00) | 0.23±0.00 (0.02±0.00) | 0.33±0.02 (0.14±0.02) | 0.00±0.00 (0.00±0.00) | 0.09±0.00 (0.04±0.01) | 0.00±0.00 (0.00±0.00) |
| Maltose | 4.84±0.00 (0.53±0.00) | 9.19±0.14 (0.93±0.05) | 63.91±1.13 (1.44±0.11) | 62.77±47.81 (1.38±0.41) | 1.80±0.05 (0.51±0.08) | 8.42±3.61 (0.61±0.14) | 1.04±0.03 (0.36±0.06) | 24.55±0.33 (1.53±0.20) |
| Mannose | 13.64±0.05 (0.94±0.00) | 14.71±0.56 (1.11±0.07) | 74.95±0.21 (1.54±0.12) | 20.80±0.88 (1.07±0.20) | 3.39±0.07 (0.73±0.10) | 15.55±0.49 (0.97±0.11) | 2.39±0.03 (0.62±0.11) | 3.53±0.23 (0.53±0.06) |
| Rhamnose | 0.47±0.02 (0.08±0.00) | 0.03±0.01 (0.01±0.00) | 0.15±0.00 (0.01±0.00) | 0.04±0.00 (0.00±0.00) | 0.20±0.01 (0.08±0.01) | 0.12±0.01 (0.03±0.01) | 0.66±0.03 (0.26±0.04) | 0.12±0.01 (0.03±0.00) |
|  |  |  |  |  |  |  |  |  |
| Ribose | 36.07±0.81 (1.42±0.01) | 40.71±1.01 (1.52±0.09) | 75.43±56.34 (1.20±0.35) | 45.20±0.18 (1.64±0.30) | 28.01±0.41 (1.75±0.25) | 33.37±0.64 (1.31±0.09) | 197.70±149.37 (2.28±0.26) | 39.21±1.47 (1.82±0.22) |
| Sorbitol | 1.16±0.03 (0.18±0.00) | 1.96±0.08 (0.42±0.03) | 14.45±0.19 (0.69±0.05) | 5.15±0.27 (0.39±0.08) | 5.20±0.41 (0.91±0.12) | 7.69±0.23 (0.69±0.11) | 2.73±0.03 (0.67±0.11) | 3.95±0.39 (0.58±0.10) |
| Sucrose | 0.17±0.00 (0.03±0.00) | 0.34±0.02 (0.11±0.01) | 0.14±0.00 (0.00±0.00) | 0.29±3.23 (2.10±0.42) | 0.29±0.02 (0.12±0.01) | 0.88±0.02 (0.16±0.05) | 0.93±0.02 (0.33±0.05) | 2.75±0.02 (0.44±0.05) |
| Xylose | 0.00±0.00 | 0.00±0.00 | 0.00±0.00 | 0.00±0.00 | 0.00±0.00 | 0.00±0.00 | 0.00±0.00 | 0.00±0.00 |
|  |  |  |  |  |  |  |  |  |

**Supplementary Table 5: Profiling of flavonoids with original mean value concentrations (µg/g)** ±**standard error values and normalized mean values**± **standard error values by LCMS method between two susceptible and two resistant parents**

| **Flavonoids** | **Mean values (µg/g) - Control** | | | | **Mean values (µg/g) -Infested** | | | |
| --- | --- | --- | --- | --- | --- | --- | --- | --- |
|  | **Susceptible** | | **Resistant** | |  | | **Resistant** | |
|  |  |  |  |  | **Susceptible** | |  |  |
|  | **IIHR 3455** | **IIHR 4604** | **IIHR 4550** | **IIHR -B-HP-79** | **IIHR 3455** | **IIHR 4604** | **IIHR 4550** | **IIHR -B-HP-79** |
| Apigenin | 0.14±0.01 (0.21±0.03) | 0.20±0.02 (0.50±0.01) | 0.30±0.01 (0.28±0.03) | 0.85±0.18 (1.14±0.24) | 0.21±0.01 (0.31±0.06) | 0.85±0.19 (1.21±0.27) | 0.16±0.01 (0.22±0.01) | 0.23±0.06 (0.40±0.19) |
| Catechin | 3.71±0.34 (2.04±0.06) | 3.75±0.04 (1.36±0.06) | 1.14±0.22 (2.33±0.10) | 2.68±0.65 (2.30±0.17) | 2.32±0.22 (1.72±0.15) | 0.85±0.11 (1.21±0.04) | 2.07±0.22 (1.57±0.09) | 14.87±13.91 (1.91±0.61) |
| Epicatechin | 4.87±0.24 (2.32±0.09) | 4.13±0.56 (2.45±0.17) | 1.90±0.42 (2.43±0.09) | 2.30±0.33 (2.16±0.04) | 4.04±0.24 (2.28±0.05) | 4.15±0.13 (3.10±0.15) | 4.50±0.09 (2.37±0.03) | 2.57±0.40 (1.92±0.52) |
| Epigallocatechin | 1.94±0.25 (1.47±0.11) | 2.03±0.31 (1.81±0.17) | 0.00±0.12 (1.66±0.08) | 1.32±0.40 (1.49±0.27) | 1.28±0.58 (1.12±0.33) | 1.24±0.30 (1.52±0.19) | 2.20±0.16 (1.63±0.06) | 1.85±0.35 (1.64±0.49) |
| Eriodictyol | 0.02±0.00 (0.03±0.00) | 0.00±0.00 (0.00±0.00) | 0.00±0.00 (0.00±0.00) | 0.01±0.00 (0.02±0.00) | 0.00±0.00 (0.01±0.00) | 0.00±0.00 (0.00±0.00) | 0.00±0.00 (0.00±0.00) | 0.00±0.00 (0.00±0.00) |
| Fisetin | 0.00±0.00 (0.00±0.00) | 0.00±0.00 (0.00±0.00) | 0.00±0.00 (0.00±0.00) | 0.00±0.00 (0.00±0.00) | 0.00±0.00 (0.00±0.00) | 0.00±0.00 (0.00±0.00) | 0.00±0.00 (0.00±0.00) | 0.00±0.00 (0.00±0.00) |
| Hesperetin | 0.38±0.05 (0.50±0.09) | 0.31±0.10 (0.69±0.11) | 0.45±0.03 (0.41±0.04) | 0.40±0.06 (0.62±0.08) | 0.29±0.09 (0.41±0.15) | 0.33±0.03 (0.58±0.01) | 0.43±0.02 (0.52±0.03) | 0.30±0.07 (0.42±0.10) |
| Kaemperol | 0.02±0.00 (0.03±0.00) | 0.03±0.00 (0.06±0.01) | 0.03±0.00 (0.04±0.01) | 0.05±0.02 (0.09±0.03) | 0.08±0.00 (0.13±0.03) | 0.03±0.00 (0.07±0.01) | 0.04±0.00 (0.06±0.00) | 0.31±0.28 (0.23±0.16) |
| Luteolin | 4.59±0.38 (2.25±0.04) | 3.27±0.52 (2.01±0.27) | 2.27±0.22 (2.21±0.09) | 3.25±0.60 (2.61±0.08) | 5.45±0.68 (2.61±0.09) | 1.27±0.27 (1.56±0.16) | 6.56±0.16 (2.79±0.08) | 14.64±11.53 (2.68±0.22) |
| Myricetin | 6.15±0.39 (2.56±0.08) | 3.50±0.59 (2.96±0.09) | 5.13±0.23 (2.24±0.04) | 2.65±0.23 (2.38±0.09) | 4.78±0.17 (2.47±0.07) | 3.45±0.58 (2.80±0.15) | 4.61±0.39 (2.38±0.06) | 3.08±0.15 (2.07±0.43) |
| Naringenin | 1.38±0.09 (1.21±0.08) | 0.97±0.09 (1.24±0.10) | 0.97±0.20 (1.01±0.10) | 1.42±0.11 (1.44±0.19) | 1.16±0.33 (1.10±0.14) | 0.98±0.02 (1.35±0.10) | 0.89±0.10 (0.91±0.06) | 0.70±0.16 (0.82±0.16) |
| Quercetin | 3.57±0.18 (2.02±0.07) | 1.61±0.42 (1.86±0.22) | 1.96±0.08 (1.45±0.04) | 1.76±0.29 (1.86±0.20) | 3.03±0.18 (1.99±0.01) | 1.27±0.19 (1.58±0.08) | 2.05±0.22 (1.57±0.09) | 1.32±0.15 (1.30±0.25) |
| Rutin | 0.67±0.22 (0.72±0.16) | 2.72±0.24 (1.13±0.17) | 0.91±0.14 (1.96±0.06) | 1.38±0.35 (1.56±0.32) | 1.90±0.12 (1.54±0.05) | 1.06±0.02 (1.43±0.09) | 1.93±0.10 (1.52±0.05) | 1.82±0.48 (1.47±0.20) |
| Umbelliferone | 0.01±0.00 (0.02±0.01) | 0.02±0.01 (0.04±0.01) | 0.02±0.00 (0.03±0.01) | 0.02±0.01 (0.04±0.01) | 0.01±0.00 (0.02±0.00) | 0.04±0.01 (0.09±0.02) | 0.01±0.00 (0.02±0.00) | 0.01±0.00 (0.02±0.00) |
| Galangin | 0.02±0.00 (0.03±0.00) | 0.03±0.00 (0.05±0.00) | 0.03±0.00 (0.04±0.01) | 0.02±0.00 (0.04±0.01) | 0.02±0.00 (0.03±0.01) | 0.02±0.00 (0.04±0.00) | 0.02±0.00 (0.03±0.00) | 0.03±0.00 (0.05±0.02) |

**Supplementary Table 6: Profiling of phenolic acids with original mean value concentrations (ng/g)** ±**standard error values and normalized mean values**± **standard error values by LCMS method between two susceptible and two resistant parents**

| **Phenolic acids** | **Mean values (ng/g) - Control** | | | | **Mean values (ng/g) -Infested** | | | |
| --- | --- | --- | --- | --- | --- | --- | --- | --- |
|  | **Susceptible** | | **Resistant** | | **Susceptible** | | **Resistant** | |
|  | **IIHR 3455** | **IIHR 4604** | **IIHR 4550** | **IIHR -B-HP-79** | **IIHR 3455** | **IIHR 4604** | **IIHR 4550** | **IIHR -B-HP-79** |
| 2,4-dihydroxybenzoic acid | 9.51±3.33 (0.63±0.13) | 5.04±1.65 (0.27±0.08) | 8.26±1.61 (0.55±0.03) | 3.43±0.20 (0.16±0.01) | 7.05±0.73 (0.63±0.06) | 4.64±0.53 (0.36±0.03) | 7.66±1.92 (0.30±0.05) | 25.80±1.07 (0.55±0.03) |
| 3-hydroxy benzoic acid | 52.49±2.53 (1.59±0.01) | 178.85±12.09 (2.18±0.04) | 56.55±1.04 (1.54±0.05) | 470.01±7.29 (2.62±0.02) | 189.75±4.09 (2.49±0.02) | 168.18±6.10 (1.94±0.09) | 653.51±34.44 (2.14±0.03) | 670.76±9.40 (2.67±0.06) |
| Benzoic acid | 279.51±16.64 (2.68±0.03) | 278.69±11.14 (2.52±0.04) | 538.69±10.34 (3.00±0.04) | 724.85±17.75 (2.93±0.02) | 156.76±12.38 (2.37±0.04) | 1539.60±25.49 (2.99±0.01) | 1362.87±75.69 (2.50±0.03) | 926.14±17.74 (2.92±0.04) |
| Caffeic acid | 4.24±0.33 (0.38±0.02) | 17.85±0.40 (0.72±0.01) | 3.45±0.23 (0.29±0.04) | 28.30±0.93 (0.81±0.01) | 1.22±0.36 (0.16±0.04) | 0.93±0.06 (0.09±0.01) | 64.17±2.02 (1.05±0.03) | 12.99±0.31 (0.32±0.00) |
| Chlorogenic acid | 738.75±20.81 (3.33±0.00) | 695.67±45.84 (3.22±0.01) | 639.54±39.77 (3.11±0.02) | 55.41±2.73 (1.17±0.03) | 361.59±23.95 (2.90±0.04) | 1691.61±55.59 (3.20±0.14) | 5191.26±453.83 (3.16±0.05) | 22.05±2.06 (0.49±0.05) |
| Ellagic acid | 28.96±6.46 (1.21±0.10) | 59.31±7.64 (1.40±0.09) | 74.14±2.12 (1.70±0.04) | 97.44±16.54 (1.51±0.11) | 48.72±4.24 (1.63±0.04) | 86.85±5.60 (1.60±0.11) | 42.36±2.12 (0.87±0.03) | 45.18±9.58 (0.79±0.11) |
| Ferulic acid | 0.59±0.02 (0.07±0.01) | 5.83±0.21 (0.31±0.01) | 1.45±0.10 (0.14±0.02) | 2.78±0.17 (0.13±0.01) | 0.47±0.14 (0.06±0.02) | 1.37±0.39 (0.13±0.04) | 2.31±0.10 (0.12±0.00) | 1.25±0.31 (0.04±0.01) |
| Gallic acid | 1.64±0.22 (0.17±0.01) | 0.00±0.00 (0.00±0.00) | 0.78±0.19 (0.08±0.02) | 3.02±0.65 (0.14±0.03) | 1.01±0.13 (0.13±0.01) | 0.78±0.11 (0.08±0.01) | 0.78±0.13 (0.04±0.01) | 36.85±2.61 (0.71±0.04) |
| Gentisic acid | 0.90±0.07 (0.10±0.01) | 1.21±0.25 (0.08±0.01) | 1.07±0.22 (0.11±0.03) | 0.48±0.06 (0.02±0.00) | 0.78±0.12 (0.11±0.02) | 4.73±0.30 (0.36±0.03) | 1.19±0.05 (0.06±0.01) | 0.67±0.19 (0.02±0.01) |
| O-coumaric acid | 0.93±0.38 (0.10±0.03) | 0.00±0.00 (0.00±0.00) | 4.59±0.41 (0.36±0.02) | 0.91±0.17 (0.05±0.01) | 0.79±0.22 (0.10±0.02) | 3.16±0.44 (0.27±0.04) | 2.02±0.18 (0.10±0.01) | 1.11±0.17 (0.03±0.00) |
| P-coumaric acid | 5.39±0.41 (0.45±0.03) | 36.60±1.25 (1.11±0.02) | 19.86±2.64 (0.94±0.07) | 7.45±0.76 (0.31±0.03) | 2.97±0.30 (0.34±0.03) | 7.20±0.72 (0.48±0.03) | 12.85±0.80 (0.45±0.01) | 31.49±6.53 (0.63±0.09) |
| P-hydroxy benzoic acid | 20.54±1.26 (1.04±0.01) | 97.55±3.56 (1.75±0.03) | 13.35±0.62 (0.76±0.03) | 283.40±14.67 (2.25±0.03) | 22.93±0.90 (1.20±0.03) | 51.75±1.25 (1.33±0.06) | 290.35±7.71 (1.75±0.02) | 549.64±91.15 (2.48±0.11) |
| Protocatechuic acid | 0.45±0.15 (0.05±0.01) | 0.42±0.05 (0.03±0.00) | 0.29±0.04 (0.03±0.00) | 0.16±0.04 (0.01±0.00) | 0.40±0.03 (0.06±0.01) | 0.31±0.04 (0.03±0.01) | 0.50±0.09 (0.03±0.01) | 0.23±0.06 (0.01±0.00) |
| Salicylic acid | 9.03±2.12 (0.63±0.07) | 0.00±0.00 (0.00±0.00) | 32.59±5.46 (1.20±0.15) | 21.59±0.82 (0.68±0.01) | 33.11±3.48 (1.40±0.07) | 71.56±3.14 (1.49±0.05) | 0.00±0.00 (0.00±0.00) | 77.69±22.35 (1.08±0.16) |
| Sinapic acid | 1.76±0.45 (0.18±0.03) | 1.75±0.16 (0.11±0.01) | 3.32±0.06 (0.28±0.03) | 4.53±1.10 (0.20±0.05) | 3.38±0.30 (0.38±0.04) | 2.42±0.16 (0.22±0.02) | 1.51±0.34 (0.08±0.02) | 13.72±1.03 (0.34±0.03) |
| Syringic acid | 0.81±0.27 (0.09±0.03) | 1.97±0.05 (0.12±0.00) | 1.13±0.10 (0.11±0.02) | 1.92±0.39 (0.09±0.02) | 1.87±0.52 (0.23±0.05) | 1.58±0.05 (0.15±0.01) | 1.33±0.23 (0.07±0.01) | 1.92±0.45 (0.06±0.01) |
| T-cinnamic acid | 175.31±7.42 (2.37±0.04) | 74.45±4.63 (1.56±0.03) | 169.51±10.39 (2.23±0.03) | 316.11±14.23 (2.33±0.03) | 179.05±7.37 (2.45±0.01) | 175.24±18.00 (1.96±0.11) | 284.42±8.63 (1.74±0.03) | 457.37±16.26 (2.37±0.02) |
| Vanillic acid | 7.49±0.29 (0.57±0.03) | 19.75±1.75 (0.77±0.04) | 4.21±1.05 (0.33±0.03) | 41.96±1.38 (1.01±0.01) | 7.13±2.13 (0.61±0.11) | 5.49±0.31 (0.41±0.03) | 10.05±1.24 (0.38±0.04) | 100.99±2.98 (1.28±0.02) |

**Supplementary Table 7: Epicuticular wax profiling of two resistant and susceptible accessions through GCMS – Liquid Injection Method**

| **Compound name** | **Retension time (min)** | **Peak area values** | | | | **P -value** | **FDR value** |
| --- | --- | --- | --- | --- | --- | --- | --- |
|  |  | **Susceptible** | | **Resistant** | |  |  |
|  |  | **IIHR 3455** | **IIHR 4604** | **IIHR 4550** | **IIHR -B-HP-79** |  |  |
| 1,2-Benzenedicarboxylic acid, bis(2-methylpropyl) ester | 18.934 | 0 | 2237400 | 0 | 0 | * | 0.00 |
| 1,4-Dibutyl benzene-1,4-dicarboxylate | 21.237 | 0 | 3740047.67 | 3293634 | 0 | * | 0.01 |
| 1-Heptacosanol | 26.191 | 3625062 | 7394485.67 | 0 | 0 | * | 0.00 |
| 1-Nonadecene | 22.148 | 4842326 | 5560260 | 5508346 | 8444594 | * | 0.03 |
| 2,4-Di-tert-butylphenol | 7.708 | 0 | 1747080 | 0 | 0 | * | 0.00 |
| 2-Hydroxy-3-isopropyl-5-piperidinomethyl-2,4,6-cycloheptatrien-1-one | 41.465 | 0 | 1991611.67 | 0 | 0 | * | 0.00 |
| Ascorbyl Palmitate | 28.595 | 0 | 0 | 5014933 | 0 | * | 0.01 |
| Dibutyl phthalate | 20.116 | 4811784 | 5221269 | 4935994 | 4829102 | NS | 0.00 |
| Eicosanoic acid, 2-[(1-oxohexadecyl)oxy]-1-[[(1-oxohexadecyl)oxy]methyl]ethyl ester | 35.962 | 0 | 0 | 0 | 56414514 | * | 0.01 |
| Heptadecyl alcohol | 42.11 | 0 | 0 | 0 | 40304865 | * | 0.00 |
| Hexacontane | 50.54 | 0 | 0 | 2132250 | 0 | * | 0.00 |
| Hexadecanoic acid, 2-[(1-oxotetradecyl)oxy]-1,3-propanediyl ester | 52.168 | 12379163 | 0 | 0 | 46371362 | * | 0.00 |
| Hexatriacontane | 44.436 | 1.18E+08 | 1606705.67 | 3153665 | 0 | * | 0.01 |
| Laurin, 2-capri-1,3-di- | 54.9 | 46917321 | 0 | 8728044 | 0 | NS | 0.00 |
| N,N-Dipropylacetamide, benzene-1,2-dioxybis- | 37.989 | 4409032 | 2586083.67 | 0 | 0 | * | 0.01 |
| Octacosyl acetate | 47.56 | 1534441 | 0 | 0 | 9986788 | * | 0.00 |
| Octadecanoic acid, (2,2-dimethyl-1,3-dioxolan-4-yl)methyl ester | 27.46 | 1.32E+08 | 0 | 3321843 | 0 | NS | 0.00 |
| Octadecanoic acid, 3-[(1-oxohexadecyl)oxy]-2-[(1-oxotetradecyl)oxy]propyl ester | 37.75 | 1.31E+08 | 0 | 29179841 | 1.76E+08 | NS | 0.00 |
| Phenol, 4-(2-methylpropyl)- | 14.795 | 9780415 | 0 | 4151591 | 0 | NS | 0.00 |
| Tetrapentacontane | 50.54 | 1659984 | 9798687 | 0 | 0 | * | 0.03 |
| Tritriacontan-one | 34.099 | 0 | 0 | 8739148 | 0 | * | 0.00 |
| 1-Hexacosanol | 33.656 | 0 | 3333726 | 2838530 | 4181570 | * | 0.00 |
| Tripalmitin | 38.082 | 1.52E+08 | 0 | 1.93E+08 | 0 | * | 0.02 |
| Tris(2,4-di-tert-butylphenyl) phosphate | 63.058 | 0 | 5098968 | 0 | 0 | * | 0.00 |
| Pentacosane | 52.965 | 13227096 | 4276895 | 0 | 16035014 | * | 0.00 |
| Phenylpropionic acid pentafluorobenzyl ester | 22.46 | 4603321 | 0 | 3840985 | 0 | * | 0.02 |
| Octadecanoic acid, 1-[[(1-oxohexadecyl)oxy]methyl]-1,2-ethanediyl ester | 41.327 | 3.96E+08 | 0 | 3031849 | 0 | NS | 0.00 |
| Hexadecanoic acid, 2-hydroxy-1,3-propanediyl ester | 34.693 | 83732745 | 1041099 | 3910967 | 0 | NS | 0.00 |
| Hexadecanoic acid, 1-[(hexadecyloxy)methyl]-1,2-ethanediyl ester | 55.835 | 29341245 | 0 | 23370388 | 4063369 | NS | 0.00 |
| Bis(2-ethylhexyl) phthalate | 32.205 | 4979952 | 435612 | 0 | 0 | * | 0.00 |
| Decanoic acid, 1,2,3-propanetriyl ester | 44.08 | 0 | 0 | 744442 | 22774305 | * | 0.00 |
| Docosanoic acid, 2-hydroxy-, methyl ester | 54.05 | 0 | 0 | 7196036 | 11743326 | * | 0.00 |
| Dodecanoic acid, 1,2,3-propanetriyl ester | 52.449 | 17542256 | 5596983 | 0 | 0 | * | 0.00 |

(*- Significance at 0.05 level; **- Significance at 0.01 level; *** - Significance at 0.001 level; Peak area values are log transformed; FDR- False Discovery Rate; P- values are based on ANOVA)

**Supplementary Table 8: Leaf volatile profiling of two resistant and susceptible parents through GCMS – SPME Method**

| **Compound name** | **Retention time (min)** | **Peak area values** | | | | | |
| --- | --- | --- | --- | --- | --- | --- | --- |
|  |  | **Susceptible** | | **Resistant** | | **P -value** | **FDR value** |
|  |  | **IIHR 3455** | **IIHR 4604** | **IIHR 4550** | **IIHR -B-HP-79** |  |  |
| 2-Hexen-1-ol, propanoate | 22.496 | 92562.33 | 67435.33 | 69028.33 | 42467.67 | *** | 0.00 |
| 3-Buten-2-ol | 5.66 | 74662.33 | 67682.33 | 68456.33 | 46040.33 | NS | 0.00 |
| 3-Hexen-1-ol formate | 16.735 | 92562.33 | 68563.33 | 68745.33 | 42467.67 | NS | - |
| 4-Hexen-1-ol, 2-methylpropionate | 27.462 | 151897 | 96797.33 | 98733.67 | 52190.67 | NS | - |
| Butyrate hexyl- | 26.986 | 0 | 0 | 2383582 | 3001286 | *** | 0.00 |
| Furan, 2-ethyl- | 11.472 | 56987.67 | 59186.67 | 60516.33 | 59900.33 | NS | - |
| Hex-2(E)-enal | 8.515 | 351267.3 | 1559761 | 549036.7 | 441082.7 | NS | - |
| Hex-2(E)-enol | 9.197 | 628205.7 | 661539 | 619973.7 | 573358.3 | NS | - |
| Hex-2(E)-enyl butyrate | 27.128 | 0 | 0 | 2203670 | 3833570 | *** | 0.00 |
| Hex-3(Z)-enol | 8.696 | 884255.3 | 917664.7 | 663244.3 | 675294 | NS | 0.00 |
| Hex-3(Z)-enyl acetate | 16.762 | 9559993 | 9363529 | 712685.3 | 351479.3 | *** | 0.00 |
| Hex-3(Z)-enyl butyrate | 26.65 | 0 | 0 | 1884905 | 3444298 | *** | 0.00 |
| Oxalic acid, allyl butyl ester | 5.465 | 26120.33 | 35633.33 | 26120.33 | 16457 | ** | 0.01 |
| Phthalic anhydride | 32.981 | 172037.7 | 187904 | 133915 | 150147.3 | NS | - |
| Ethanoate hexyl | 17.163 | 16073.7 | 13336.7 | 16073.7 | 0.0 | *** | 0.01 |

(*-Significance at 0.05 level; **- Significance at 0.01 level; *** - Significance at 0.001 level; Peak area values are log transformed; FDR- False Discovery Rate; P- values are based on ANOVA)

**Supplementary Table 9: Untargeted leaf volatile profiling of two resistant and susceptible parents through GCMS – Liquid Injection Method**

| **Serial No** | **Functional Group** | **Compound Name** | **Retention Time (min)** | **CAS Number** | **% Peak area values** | | | |
| --- | --- | --- | --- | --- | --- | --- | --- | --- |
|  |  |  |  |  | **IIHR 3455 (S)** | **IIHR 4604 (S)** | **IIHR 4550 (R)** | **IIHR-B-HP-79 (R)** |
| 1 | Acids | Chloromethyl chloroacetate | 4.593 | 6135-23-5 | 0.001 | 0.001 | 0.001 | 0.001 |
| 2 | Alcohols | Propargyl alcohol | 5.477 | 107-19-7 | 0.001 | 0.001 | 0.11 | 0.08 |
| 3 |  | Hexanol (n-) | 17.167 | 111-27-3 | 0.065 | 0.315 | 0.46 | 0.001 |
| 4 |  | 3-Buten-2-ol | 5.66 | 598-32-3 | 2.001 | 1.16 | 1.191 | 0.34 |
| 5 |  | 3-Penten-2-ol | 21.611 | 1569-50-2 | 0.001 | 0.001 | 0.79 | 0.001 |
| 6 |  | Hex-3(Z)-enol | 8.722 | 928-96-1 | 15.48 | 30.48 | 6.09 | 8.81 |
| 7 |  | Hex-2(E)-enol | 9.218 | 928-95-0 | 2.64 | 6.225 | 7.43 | 2.95 |
| 8 | Aldehydes | Sorbic aldehyde | 11.392 | 142-83-6 | 0.001 | 0.84 | 0.61 | 0.001 |
| 9 |  | 4-Oxohex-2-enal | 14.166 | 20697-55-6 | 0.001 | 0.945 | 0.001 | 0.001 |
| 10 |  | Hexanal | 6.247 | 66-25-1 | 0.001 | 1.085 | 0.001 | 0.001 |
| 11 |  | Hex-2(E)-enal | 8.538 | 6728-26-3 | 1.25 | 12.365 | 5.52 | 1.91 |
| 12 |  | 3-Hexenal | 6.222 | 4440-65-7 | 0.18 | 0.001 | 0.001 | 0.001 |
| 13 | Alkanes | Hexane, 2,2,3-trimethyl- | 4.143 | 16747-25-4 | 0.001 | 0.001 | 0.43 | 0.001 |
| 14 |  | 1-Ethoxy-3-methyl-2-butene | 36.618 | 0-00-0 | 0.001 | 0.001 | 0.44 | 0.001 |
| 15 |  | 1-Heptene, 1,3-diphenyl-1-(trimethylsilyloxy)- | 40.328 | 0-00-0 | 0.001 | 0.001 | 0.08 | 0.001 |
| 16 | Alkynes | 1-Butyne, 3,3-dimethyl- | 4.427 | 917-92-0 | 0.001 | 0.001 | 0.01 | 0.001 |
| 17 | Amines | 2-Amino-1,3,5-triazine | 11.052 | 04-07-4122 00:00 | 0.001 | 0.14 | 0.001 | 0.001 |
| 18 |  | 4-(2-amino-1-hydroxypropyl)phenol, 2TMS derivative | 24.132 | 0-00-0 | 0.001 | 0.6 | 0.001 | 0.001 |
| 19 |  | 5-Amino-1-methyl-1H-pyrazole-4-carboxamide, 3TMS | 32.645 | 0-00-0 | 0.001 | 0.15 | 0.001 | 0.001 |
| 20 |  | 1-Phenylsulphonylamino-4,5-diphenyl-triazole | 39.684 | 120675-07-2 | 0.001 | 0.055 | 0.001 | 0.001 |
| 21 |  | 2-Aminocyanoacetamide | 10.152 | 6719-21-7 | 0.001 | 0.001 | 0.001 | 0.035 |
| 22 |  | 5-Amino-1-methyl-1H-pyrazole-4-carboxamide, 3TMS | 32.681 | 0-00-0 | 0.07 | 0.001 | 0.001 | 0.001 |
| 23 | Amino Acids | dl-Threonine | 4.135 | 80-68-2 | 0.001 | 0.35 | 0.001 | 0.001 |
| 24 | Anhydrides | Phthalic anhydride | 32.999 | 85-44-9 | 1.58 | 1.65 | 0.47 | 0.91 |
| 25 |  | Trifluoromethanesulfonic anhydride | 22.837 | 358-23-6 | 0.001 | 0.001 | 0.12 | 0.001 |
| 26 | Aromatic Amines | Benzeneethanamine, N-[(pentafluorophenyl)methylene]-.beta.,4-bis[(trimethylsilyl)oxy]- | 24.179 | 55429-85-1 | 0.001 | 0.001 | 0.07 | 0.001 |
| 27 |  | Oxybenzene | 4.194 | 108-95-2 | 0.001 | 0.96 | 0.001 | 0.001 |
| 28 | Boron Compounds | Borane, ethylisopropylmethyl- | 8.493 | 0-00-0 | 0.09 | 0.001 | 0.001 | 0.001 |
| 29 | Carboxylic Acids | Pentanoic acid | 6.083 | 109-52-4 | 0.001 | 0.001 | 0.59 | 0.77 |
| 30 |  | 3-Penten-1-yne, 3-methyl- | 17.782 | 1574-33-0 | 0.57 | 0.001 | 0.001 | 0.001 |
| 31 |  | 2-Butenoic acid, 2,3-dimethyl- | 36.629 | 4411-97-6 | 0.001 | 0.001 | 0.21 | 0.7 |
| 32 |  | 1-Methylcyclohexylcarboxylic acid | 14.462 | 1123-25-7 | 0.001 | 0.001 | 0.16 | 0.001 |
| 33 |  | 2-Hexenoic acid, methyl ester, (E)- | 14.478 | 13894-63-8 | 0.001 | 0.001 | 0.55 | 0.001 |
| 34 |  | Butanoic acid, 2-pentenyl ester, (Z)- | 21.634 | 42125-13-3 | 0.001 | 0.001 | 0.6 | 0.001 |
| 35 |  | 2-Butenoic acid, butyl ester | 29.603 | 7299-91-4 | 0.001 | 0.001 | 0.06 | 0.001 |
| 36 | Cyclopropanes | Cyclopropane, propyl- | 9.372 | 2415-72-7 | 0.001 | 0.001 | 0.54 | 0.001 |
| 37 |  | Ethanone, 1-(2-methylcyclopropyl)- | 36.821 | 930-56-3 | 0.001 | 0.001 | 0.11 | 0.001 |
| 38 | Dinitriles | 2-{Amino[2-cyano-3-(2-nitrophenyl)oxiran-2-yl]methylidene}propanedinitrile | 15.64 | 0-00-0 | 0.001 | 0.001 | 0.08 | 0.001 |
| 39 | Epoxides | 2-Methyl-3,4-epoxy-1-butene | 4.878 | 0-00-0 | 0.001 | 0.18 | 0.24 | 0.001 |
| 40 | Esters | Butanoic acid, methyl ester | 4.167 | 623-42-7 | 0.001 | 0.001 | 0.001 | 0.62 |
| 41 |  | Butyrate (ethyl) | 6.323 | 105-54-4 | 0.001 | 0.001 | 0.001 | 0.76 |
| 42 |  | Lactate (cis-3-hexenyl) | 17.306 | 61931-81-5 | 0.215 | 0.001 | 0.76 | 0.34 |
| 43 |  | 2-Hexen-1-ol, propanoate | 22.496 | 53398-80-4 | 1.34 | 1.25 | 1.27 | 1.03 |
| 44 |  | Hex-3(Z)-enol propanoate | 28.99 | 33467-74-2 | 0.07 | 0.001 | 0.775 | 0.535 |
| 45 |  | 3-Hexen-1-ol, formate | 16.735 | 33467-73-1 | 2.25 | 0.95 | 1.1201 | 0.68 |
| 46 |  | Hex-3(Z)-enyl butyrate | 26.656 | 16491-36-4 | 0.525 | 0.001 | 20.63 | 36.905 |
| 47 |  | Butyrate (hexyl) | 26.986 | 2639-63-6 | 0.001 | 0.001 | 12.605 | 14.93 |
| 48 |  | Hex-2(E)-enyl butyrate | 27.137 | 53398-83-7 | 0.001 | 0.001 | 16.465 | 19.27 |
| 49 |  | Propanoic acid, butyl ester | 22.352 | 590-01-2 | 0.001 | 0.001 | 0.001 | 0.22 |
| 50 |  | 4-Hexen-1-ol, 2-methylpropionate | 27.462 | 0-00-0 | 0.03 | 1.6041 | 1.74 | 0.69 |
| 51 |  | (E)-But-2-en-1-yl 2-methylbutanoate | 29.296 | 0-00-0 | 0.001 | 0.001 | 0.001 | 0.3 |
| 52 |  | Hex-3(Z)-enyl acetate | 16.765 | 3681-71-8 | 74.84 | 54.17 | 8.13 | 19.54 |
| 53 |  | Ethanoate (hexyl-) | 17.163 | 142-92-7 | 0.745 | 0.47 | 0.21 | 0.001 |
| 54 |  | 2-Hexen-1-ol, acetate, (E)- | 17.23 | 2497-18-9 | 0.001 | 0.2 | 0.28 | 0.001 |
| 55 |  | Acrylic acid, 3-methylene-4-pentenyl ester | 17.765 | 0-00-0 | 0.001 | 0.09 | 0.001 | 0.001 |
| 56 |  | 3,3-Diethoxy-1-propyne | 18.189 | 10160-87-9 | 0.001 | 0.16 | 0.03 | 0.001 |
| 57 |  | Oxalic acid, butyl cyclobutyl ester | 22.335 | 0-00-0 | 0.001 | 0.001 | 0.09 | 0.001 |
| 58 |  | 2-Propyn-1-ol, propionate | 22.486 | 1932-92-9 | 0.001 | 0.001 | 0.23 | 0.001 |
| 59 |  | Butanoate (2-methyl-, 3(Z)-hexenyl-, cis-) | 28.974 | 53398-85-9 | 0.001 | 0.001 | 0.09125 | 0.001 |
| 60 |  | trans-2-Hexenyl 2-methylbutyrate | 29.278 | 0-00-0 | 0.001 | 0.001 | 0.42 | 0.001 |
| 61 |  | Caproate (3(Z)-hexenyl-) | 36.236 | 31501-11-8 | 0.001 | 0.001 | 0.735 | 0.001 |
| 62 |  | Hexanoic acid, hexyl ester | 36.476 | 6378-65-0 | 0.001 | 0.001 | 0.2 | 0.001 |
| 63 |  | Propanoic acid, 2-methyl-, 2-propenyl ester | 5.368 | 15727-77-2 | 0.001 | 0.001 | 0.23 | 0.001 |
| 64 |  | Propionate (hexyl-) | 22.353 | 2445-76-3 | 0.001 | 0.001 | 0.46 | 0.001 |
| 65 |  | Hex-3(Z)-enyl isobutanoate | 24.353 | 41519-23-7 | 0.001 | 0.001 | 0.23 | 0.001 |
| 66 |  | 4-Hexen-1-ol, 2-methylpropionate | 27.466 | 0-00-0 | 0.001 | 0.001 | 0.74 | 0.001 |
| 67 |  | 4-Penten-1-ol, 2-methylene- | 27.783 | 30457-84-2 | 0.001 | 0.001 | 0.07 | 0.001 |
| 68 |  | trans-2-Hexenyl 2-methylbutyrate | 29.287 | 0-00-0 | 0.001 | 0.001 | 0.97 | 0.001 |
| 69 |  | cis-3-Hexenyl cis-3-hexenoate | 36.423 | 61444-38-0 | 0.001 | 0.001 | 0.76 | 0.001 |
| 70 |  | Oxalic acid, allyl butyl ester | 22.502 | 0-00-0 | 1.129 | 1.46 | 1.13 | 0.09 |
| 71 | Furans | Furan, 2-ethyl- | 11.472 | 3208-16-0 | 0.46 | 0.49 | 1.75 | 0.64 |
| 72 |  | 2-Furanmethanol, tetrahydro- | 5.346 | 97-99-4 | 0.26 | 0.001 | 0.001 | 0.001 |
| 73 | Imidazolidines | 1-Methyl-2,4,5-trioxoimidazolidine | 26.579 | 3659-97-0 | 0.001 | 0.19 | 0.001 | 0.001 |
| 74 | Ketones | 2-Pentyn-4-one | 28.985 | 7299-55-0 | 0.001 | 0.001 | 0.001 | 0.05 |
| 75 | Lactates | 1-Oxetan-2-one, 4-methyl-3-methylene- | 8.214 | 0-00-0 | 0.001 | 0.001 | 0.07 | 0.001 |
| 76 |  | 1-Oxetan-2-one, 4-methyl-3-methylene- | 8.15 | 0-00-0 | 0.001 | 0.14 | 0.001 | 0.001 |
| 77 | Naphthalenes | Naphthalene, 2-ethenyl- | 36.352 | 827-54-3 | 0.001 | 0.28 | 0.001 | 0.84 |
| 78 | Nitriles | Butanenitrile, 2-methyl- | 4.473 | 18936-17-9 | 0.001 | 0.001 | 0.001 | 0.03 |
| 79 |  | 2-Propynenitrile, 3-fluoro- | 22.775 | 32038-83-8 | 0.001 | 0.09 | 0.001 | 0.001 |
| 80 | Nitro Compounds | Nitric acid, butyl ester | 33.477 | 928-45-0 | 0.001 | 0.001 | 0.001 | 0.1 |
| 81 |  | 1-Phenylsulphonylamino-4,5-diphenyl-triazole | 39.747 | 120675-07-2 | 0.001 | 0.055 | 0.001 | 0.11 |
| 82 | Oxiranes | Oxirane, propyl- | 21.655 | 1003-14-1 | 0.001 | 0.001 | 0.001 | 0.28 |
| 83 | Phenolic Compounds | Methyl salicylate | 26.997 | 119-36-8 | 0.001 | 0.31 | 0.001 | 0.001 |
| 84 | Phosphoric Acids | Phosphorous acid, dimethyl ester | 27.567 | 96-36-6 | 0.001 | 0.001 | 0.56 | 0.001 |
| 85 | Phosphorus Compounds | Dimethyl phosphite | 27.589 | 868-85-9 | 0.001 | 0.001 | 0.32 | 0.12 |
| 86 | Pyrazines | Pyrazine | 4.528 | 290-37-9 | 0.001 | 0.001 | 0.001 | 0.02 |
| 87 | Pyrazoles | 5-Amino-1-methyl-1H-pyrazole-4-carboxamide, 3TMS | 32.706 | 0-00-0 | 0.001 | 0.001 | 0.32 | 0.001 |
| 88 | Pyridazines | 4-Methylpyridazine | 4.261 | 1120-88-3 | 0.001 | 0.001 | 0.001 | 0.14 |
| 89 |  | 3-Methylpyridazine | 4.251 | 1632-76-4 | 0.001 | 0.001 | 0.33 | 0.001 |
| 90 | Pyridines | Pyridine, 2,3,4,5-tetrahydro- | 36.833 | 505-18-0 | 0.001 | 0.001 | 0.56 | 0.35 |
| 91 |  | Hexahydropyridine, 1-methyl-4-[4,5-dihydroxyphenyl]- | 6.859 | 94427-47-1 | 0.001 | 0.001 | 0.16 | 0.001 |
| 92 | Silicon Compounds | p-Trimethylsilyloxyphenyl-bis(trimethylsilyloxy)ethane | 24.189 | 0-00-0 | 0.52 | 0.7601 | 1.38 | 1.07 |
| 93 |  | 5-Isopentyl-6-methyl-2-sulfanyl-4-pyrimidinol | 32.703 | 0-00-0 | 0.001 | 0.001 | 0.001 | 0.11 |
| 94 |  | Trisiloxane, octamethyl- | 39.752 | 107-51-7 | 0.001 | 0.001 | 0.36 | 0.04 |
| 95 |  | 4-(2-amino-1-hydroxypropyl)phenol, 2TMS derivative | 24.197 | 0-00-0 | 0.001 | 0.001 | 0.001 | 0.54 |
| 96 |  | 5-Isopentyl-6-methyl-2-sulfanyl-4-pyrimidinol, 2 TMS derivative | 32.712 | 0-00-0 | 1.1301 | 1.101 | 1.1301 | 1.1301 |
| 97 | Others | Cyclotetrasiloxane, octamethyl- | 15.648 | 556-67-2 | 0.001 | 0.001 | 0.76 | 0.095 |
| 98 |  | 2-[(Trimethylsilyl)oxy]-2-{4-[(trimethylsilyl)oxy]phenyl}ethanamine | 24.197 | 0-00-0 | 0.001 | 0.001 | 0.61 | 0.001 |
| 99 |  | 2,2-Dimethyl-propyl 2,2-dimethyl-propanesulfinyl sulfone | 5.381 | 82360-14-3 | 0.001 | 0.001 | 0.001 | 0.22 |
| 100 |  | Methanesulfinyl fluoride | 16.75 | 56755-41-0 | 0.21 | 0.09 | 0.43 | 0.63 |
| 101 |  | 4-Chlorobenzenesulfonamide, N-methyl- | 6.864 | 6333-79-5 | 0.001 | 0.001 | 0.09 | 0.001 |
| 102 |  | 4-Amino-5-cyclohexyl-4H-1,2,4-triazol-3-yl hydrosulfide, 2TMS derivative | 30.476 | 0-00-0 | 0.001 | 0.001 | 0.16 | 0.001 |
